# Forced vital capacity and cross-domain late-onset Pompe disease outcomes: an individual patient-level data meta-analysis

**DOI:** 10.1007/s00415-019-09401-1

**Published:** 2019-06-11

**Authors:** Kenneth I. Berger, Steve Kanters, Jeroen P. Jansen, Andrew Stewart, Susan Sparks, Kristina An Haack, Anna Bolzani, Gaye Siliman, Alaa Hamed

**Affiliations:** 1 André Cournand Pulmonary Physiology Laboratory, Bellevue Hospital, New York, NY USA; 2 Division of Pulmonary, Critical Care and Sleep Medicine, New York University, New York, NY USA; 3 Precision Xtract, Vancouver, BC Canada; 4 Precision Xtract, Oakland, CA USA; 5 Sanofi Genzyme, Cambridge, MA USA

**Keywords:** Late-onset Pompe disease, Forced vital capacity, 6-min walk test, Individual patient-level data meta-analysis

## Abstract

**Background:**

Late-onset Pompe disease (LOPD) is a rare, metabolic disease primarily affecting the musculoskeletal and respiratory systems. Forced vital capacity (FVC) is commonly used to measure pulmonary function; however, associations between FVC and other LOPD outcomes remain unclear.

**Methods:**

A systematic literature review was conducted on November 2015, updated September 2016 and supplemented with clinical trial data from the sponsor. Outcomes included: 6-min walk test distance (6MWT), FVC, maximal inspiratory/expiratory pressure (MIP/MEP), Medical Research Council-skeletal muscle strength score (MRC), 36-item short-form survey-physical component score (SF-36), Rotterdam Handicap Scale (RHS), Fatigue Severity Scale (FSS) and survival. Individual patient data meta-analysis was used for cross-sectional analyses and longitudinal analyses to determine associations between percent of predicted FVC and LOPD measures and outcomes.

**Results:**

Fifteen studies were selected. From cross-sectional analyses, FVC and MRC were most strongly associated. Specifically, patients with 10% higher FVC (a round number for illustrative purposes only) were associated with a 4.72% (95% confidence interval [CI]: 3.37, 6.07) higher MRC score, indicating a positive association. Similarly, slopes for the 6MWT and SF-36 relative to a 10% higher FVC were estimated at 33.2 meters (95% CI 24.0, 42.4) and 1.2% (95% CI 0.24, 2.16%), respectively. From longitudinal analyses, a 10% incremental increase in predicted FVC was associated with an average increase of 4.12% in MRC score (95% CI 1.29, 6.95), 35.6 m in the 6MWT (95% CI 19.9, 51.6), and 1.34% in SF-36 (95% CI 0.08, 2.60). There was insufficient data to conduct analyses for RHS, FSS and survival.

**Conclusions:**

FVC is positively associated with LOPD measures and outcomes across multiple domains. Additionally, longitudinal changes in FVC are positively associated with changes in the 6MWT, MRC and SF-36.

**Electronic supplementary material:**

The online version of this article (10.1007/s00415-019-09401-1) contains supplementary material, which is available to authorized users.

## Introduction

Late-onset Pompe disease (LOPD) is a rare metabolic disease caused by a deficiency of acid α-glucosidase (GAA) resulting in the accumulation of glycogen in primarily skeletal and cardiac muscles [1]. The accumulation of glycogen within muscles results in progressive, irreversible muscle damage, leading to muscle weakness and respiratory insufficiency [v]. With more severe disease, ventilator support and wheelchair dependency are increasingly likely, as is mortality [2].

As LOPD tends to affect the whole body, there are several outcome measures that are used to assess disease progression within an LOPD patient. Domains that have been frequently assessed include respiratory capacity, exercise capacity and endurance, symptoms (e.g., fatigue), and global assessments (e.g., quality of life). Forced vital capacity (FVC) is a measure of respiratory function that is among the more commonly reported outcomes assessed in LOPD patients as respiratory function is a key area of impact for the disease, and FVC is an objective and reproducible parameter that is readily measurable across most settings. Accordingly, FVC was a primary endpoint of the Late Onset Treatment Study and Extension (LOTS) [3, 4], as well as other studies that demonstrate the efficacy of enzyme replacement therapy (ERT) with alglucosidase alfa. Nevertheless, little is known about the association between FVC and other LOPD outcome measures, both with respect to observed values and to change within these measures.

For the present study, a literature review and meta-analysis of patient level data were performed to determine: (1) the relationship between respiratory function, as assessed by FVC, and outcomes in other domains including endurance, skeletal muscle function, physical function, symptoms and quality of life; and (2) the relationship between the changes in respiratory function and changes in other domains that occur in response to ERT.

## Methods

### Study selection

Eligible studies were those reporting individual patient-level data on LOPD patients, regardless of treatments, with FVC and at least one other LOPD outcome. Studies of any design were included except for case reports. The systematic literature review was conducted in multiple phases up to November 14, 2015. This was supplemented with a targeted review conducted in September 2016. Additionally, the evidence base was also supplemented by individual patient-level data provided by Sanofi-Genzyme from the LOTS trial and extension [3, 4]. Search strategies as well as table of inclusion criteria can be found in the Web Appendix.

Patient overlap between studies was identified by location and reported overlap. There were single Danish, French, Swiss, Greek, and Polish studies; so overlap of data from these studies was unlikely [5-9]. There were two German studies that reported data on two patients each; these were deemed to have no overlap since the studies were based in different hospitals [10, 11]. None of the Dutch studies included in these analyses suffered from patient overlap. The identification of overlap in Italian studies was the most involved. The only outcome for which multiple Italian studies were included was the 6MWT, which included four Italian studies [12-15]. However, analysis of study dates and patient characteristics suggested minimal overlap. See the Web Appendix for further details.

### Data extraction

Using a data extraction form, two investigators (SK and AB) extracted data from the materials obtained through the systematic searches. Data were reconciled to remove all discrepancies between reviewers and in case of disagreements, a third reviewer acted as an arbitrator. Some data were extracted from published graphs using the DigitizeIt software (version 15; Braunschweig, Germany). In this circumstance, data extraction was conducted by a single reviewer and then reviewed by a second reviewer. All data were stored and managed in Microsoft Excel Workbooks.

Regarding study design characteristics, the following information was extracted: study design, study inclusion/exclusion criteria, active treatment duration, follow-up period duration, sample size at baseline and follow-up by intervention, study location, study time period, and number of study sites.

The following patient characteristics were extracted: age, sex, time since diagnosis, disease duration, time from diagnosis to treatment, functional skill score, proportion requiring wheelchair use, proportion requiring ventilators (invasive or non-invasive), 6-min walk test distance (6MWT), FVC, maximal inspiratory pressure (MIP), maximal expiratory pressure (MEP), Medical Research Council-skeletal muscle strength score (MRC), Rotterdam Handicap Scale (RHS), Fatigue Severity Scale (FSS), Short-form survey with 36 items-physical component score (SF-36 PCS), Walton and Gardner-Medwin score for motor disability (WGM), Gait–Stairs–Gower–Chair (GSGC), and height.

The outcome variables were: 6MWT, MRC, RHS, FSS, SF-36 PCS, WGM, GSGC, MIP, MEP, and survival. FVC data were primarily reported as the percent of predicted normal. Given the nature of the research question, the basic model involved a selected outcome as the dependent variable with FVC as a predictor. Note, a single study reported vital capacity rather than FVC [16]. Change from baseline values was calculated when feasible if only observed values were presented and vice versa.

### Statistical analysis

**Fig. 1:**
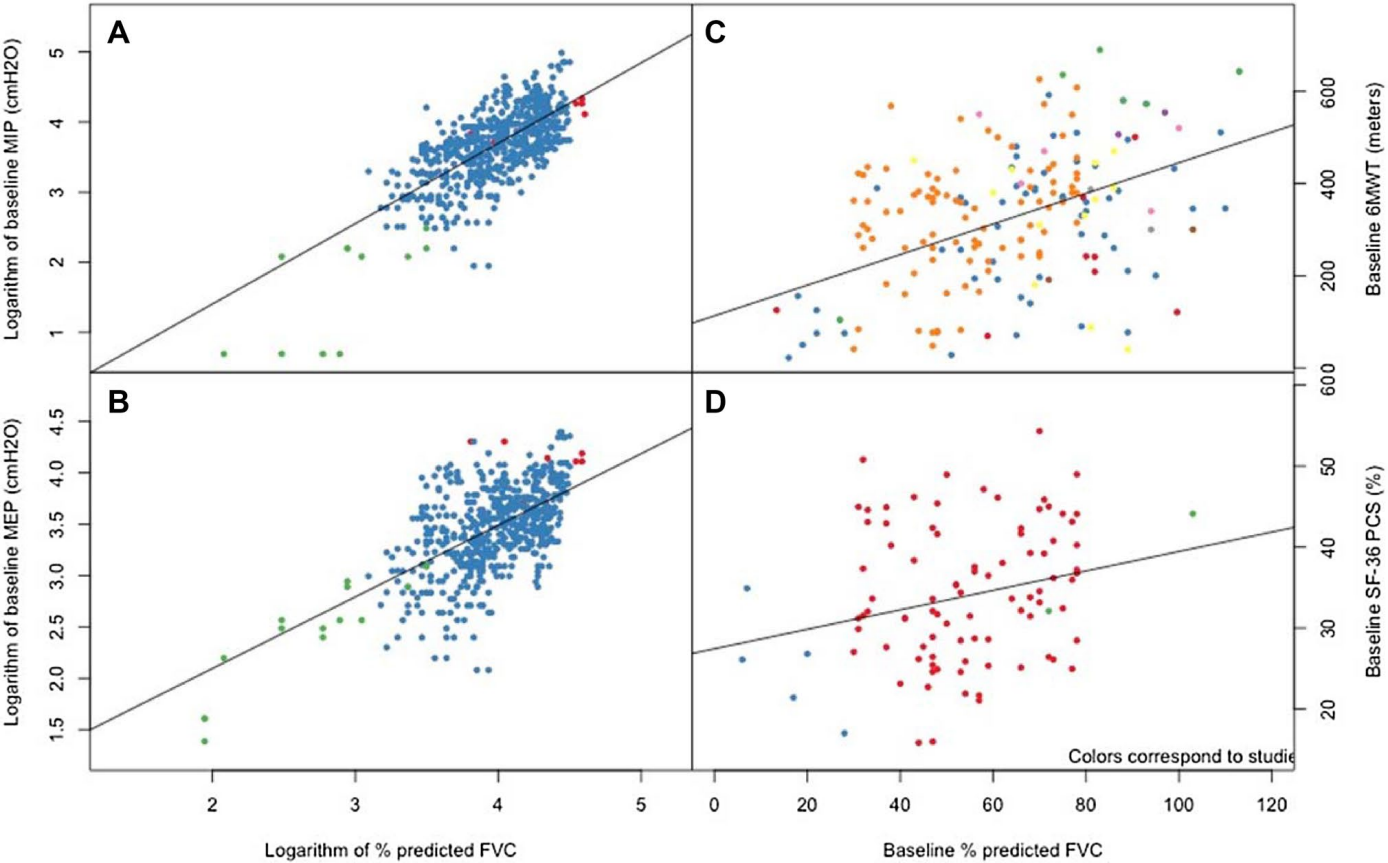
Association between logarithm-transformed baseline percent predicted FVC and logarithm-transformed baseline: **a** maximal inspiration pressure and **b** maximal expiration pressure; and baseline percent predicted FVC and baseline: **c** 6-min walk test; **d** SF-36 physical component score. Colors correspond to the studies making up the pooled cohort. In each case, the lines correspond to regression lines obtained through mixed-effects regression

Two sets of analyses were conducted. First, cross-sectional analyses were performed to explore bi-variate associations between FVC and other LOPD measures. Second, longitudinal analyses were performed to determine the association between changes in FVC and changes in LOPD outcomes. Note that only the MRC analyses included FVC and VC; all other analyses were FVC only. For both cross-sectional and longitudinal analyses, the patient-level data across studies and cohorts in the evidence base were grouped into a single large, pooled cohort. There were two sources of clustering among data points. The first level of clustering was the studies from which patients came (i.e., two patients from a single study are more likely to be similar than two patients taken from different studies), which applied to the cross-sectional and longitudinal analyses. The second level was repeated measures within individual patients, which only applied to the longitudinal analyses. Figure 1 of the Web Appendix displays the nested nature of the data graphically.

#### Cross-sectional analyses

For cross-sectional analyses, baseline values were used to avoid including multiple points from any given individual. Scatterplots were used to visualize trends and mixed-effects linear regression was used to describe the trends numerically. The mixed effects included a random effect to account for the fact that observations came from different studies. The mixed-effects models used fixed effects to describe the associations between FVC and the LOPD measures of interest.

#### Longitudinal analyses

Longitudinal data were also observed graphically through scatter plots and numerically through mixed-effects models. For outcomes with limited data (data not recorded in the LOTS study), the scatter plots were augmented to include trend lines for each individual. This approach allowed further characterization of trends both within and across patients. We used nested hierarchical modeling to account for the clustered nature of the pooled cohort with random effects used to account for both the repeated measures on individual patients and the fact that patients came from different studies [17]. For these analyses, combinations of baseline FVC, change in FVC, time, and an interaction between baseline FVC and change in FVC were used as fixed effects. For the 6MWT, we also used a dichotomous variable to identify baseline values ≥ 500 m, since this value reflects normal walking ability and treatment-induced improvement was unlikely in these patients [18]. Individual patients and studies were used as random intercepts. Data used to conduct these analyses were unbalanced; however, most patients had multiple time measurements, making it feasible to conduct generalized mixed models despite the unbalanced data.

These models were implemented in SAS 9.4 (Cary, North Carolina) using PROC MIXED. All tests were two sided and conducted at the 0.05 significance level. R 3.3.3 (Vienna, Austria) was also used for plotting.

## Results

In total, 15 studies reporting individual patient-level data were selected [3, 4, 6-8, 10-16, 19-22]. The list of studies excluded at full-text screening is presented in the Web Appendix. Included studies were published between 2008 and 2015 with average study/trial duration of 49.4 months. Of these, one study was cross-sectional [19], three studies reported case series ( ≤ 5 patients) [11, 20, 21], one was an RCT, with extension [3, 4], and ten were cohort studies (including retrospective cohorts). The evidence base contained 276 patients. The mean age was 42.2 years (SD: 16.3 years) and there were 46.5% males among trials reporting sex. Study and patient characteristics are provided in the Web Appendix. Among patients using alglucosidase alfa, time zero always represented the time of treatment initiation. Thus, change from baseline coincides with change from start of treatment. Table 1 provides information on the number of studies, patients and observations used for each analysis.

**Table 1:** Number of studies, patients and observations used for each of these analyses

	6-min walk test	SF-36 PCS	Maximal inspiratory pressure	Maximal expiratory pressure	Medical Research Council	Walton and Gardner-Medwin
*Cross-sectional analyses of observed values*						
Number of studies	10	3	3	3	4	5
Number of patients	193	97	105	104	23^a^	98^b^
*Longitudinal analyses of changes from baseline*						
Number of studies	9	3	3	3	4	5
Number of patients	176	92	95	95	19	74
Number of observations	613	374	510	508	31	80

^a^54 observations on 23 patients

^b^175 observations on 98 patients

### Cross-sectional analyses

Cross-sectional data from differing studies were combined to evaluate the relationship between FVC and other LOPD measures. There was a positive relationship between percent-predicted FVC and all LOPD measures for which there was sufficient evidence to determine a relationship. Figure 1 displays the association between percent-predicted FVC and LOPD measures. MIP and MEP were reported in individual patients along with FVC in three studies, including the LOTS and extension [3, 4, 7, 19]. As expected, baseline FVC was strongly correlated with baseline measures of both inspiratory and expiratory muscle strengths, as shown in Panels a and b of Fig. 1 which plots the logarithm of MIP and MEP (in cmH_2_O) relative to the logarithm of FVC. Applying a logarithmic transform to the MIP/MEP scores, as well as the FVC, resulted in improved linearity. The correlations, respectively, 0.761 and 0.620, were both statistically significant with *p* values < 0.0001. The 6MWT in the form of individual patient-level data was reported in 11 studies, but values in the study by Andreassen et al. were not used in the analysis because their results were reported as percent change without absolute values at baseline [3-6, 10-15, 20, 22]. Cross-sectional data relating FVC to the 6MWT (Fig. 1c) also displayed a moderate association (correlation: 0.440; *p* value < 0.0001), indicating that preservation of respiratory function was associated with better endurance. Data for FVC and SF-36 PCS were only reported in three studies; nevertheless, an association between degree of respiratory impairment and patient perception of disability and dyspnea was demonstrable (Fig. 1d), with an estimated correlation of 0.248 (*p* value < 0.0001) [3, 4, 7, 20].

**Fig. 2:**
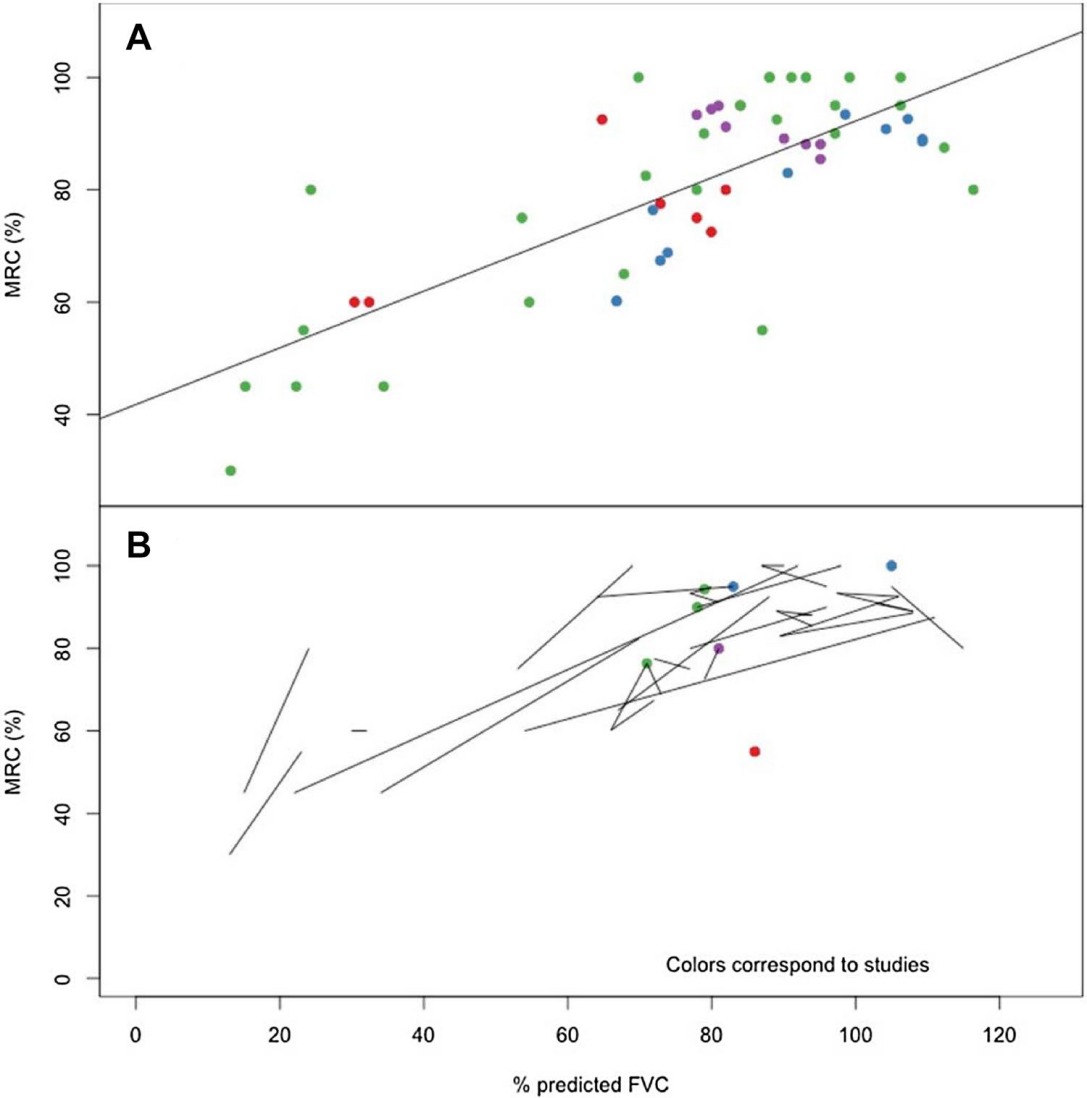
Association between percent-predicted FVC and MRC skeletal muscle strength score within the cross-sectional analyses Colors correspond to the studies making up the pooled cohort. Panel **a** displays the relationship between the observed FVC and observed MRC results with a regression line. Panel **b** displays the trajectories by individuals across time for observed values

The Medical Research Council-skeletal muscle strength score (MRC) was reported in four studies [8, 11, 16, 20]. Values were reported in different manners and reconciled by standardizing the scores to be out of 100. Figure 2 displays the associations between FVC and MRC. As there were fewer patients on which to explore the association between these two variables, data were not restricted to baseline only. As such, we were able to draw trajectories for each patient (Fig. 2b) and these suggested a consistent direct association between changes in respiratory function, as assessed by FVC, and changes in skeletal muscle strength. This association had the highest estimated correlation, 0.765 (*p* value < 0.0001).

Table 2 displays all results of the cross-sectional analyses, which accounted for the fact that observations varied with respect to study of origin. All associations between FVC and the LOPD measures described in this section were statistically significant, and in most cases, there was high likelihood of an association (i.e., *p* values for non-zero slopes < 0.0001). In addition to the strength of evidence (i.e., likelihood of association) as demonstrated by the *p* value, the models for cross-sectional analyses also suggested a moderate to strong association through the magnitude of the correlation coefficients. This was the case for the association between FVC and the 6MWT. On average, a 10% higher predicted FVC was associated with a 33.2-m increase in 6MWT distance [95% confidence interval (CI) 24.0, 42.4 m]. Note that 10% is a round number that is used for illustrative purposes only. Similarly, there were strong associations with MIP and MEP, where a 10% higher FVC at 50% predicted was associated with a MIP that was 5.96 cmH_2_O (95% CI 1.92, 17.79) higher and a MEP that was 4.09 cmH_2_O (95% CI 1.19, 12.60) higher. For MIP and MEP, the effect of a 10% shift in FVC changes according to the reference FVC due to the log–log nature of the model. The change in SF-36 associated with a similar 10% increase in FVC was estimated at 1.2% (95% CI 0.24, 2.16%). This was a weak, yet statistically significant association. The association between FVC and MRC was stronger, where a 10% higher predicted FVC was associated with a 4.72% (95% CI 3.37, 6.07) higher MRC score. There were insufficient data to conduct analyses for RHS, FSS and survival.

**Table 2:** Modelled association between FVC% and LOPD measures using cross-sectional analyses

Effect	6-min walk test		SF-36 PCS		Maximal inspiratory pressure^a^		Maximal expiratory pressure^a^		Medical Research Council	
	Estimate (SE)	*p* value	Estimate (SE)	*p* value	Estimate (SE)	*p* value	Estimate (SE)	*p* value	Estimate (SE)	*p* value
Intercept	112.92 (47.057)	0.0432	27.44 (2.863)	0.0107	− 1.39 (0.526)	0.1189	0.83 (0.439)	0.1834	48.53 (6.055)	0.0041
FVC	3.32 (0.469)	< 0.0001	0.12 (0.049)	0.0172	1.18 (0.1153)	< 0.0001	0.67 (0.107)	< 0.0001	0.47 (0.069)	< 0.0001

^a^Analyses conducted using log transforms of both the outcome and FVC

*FVC* forced vital capacity, *SE* standard error

### Longitudinal analyses

**Fig. 3:**
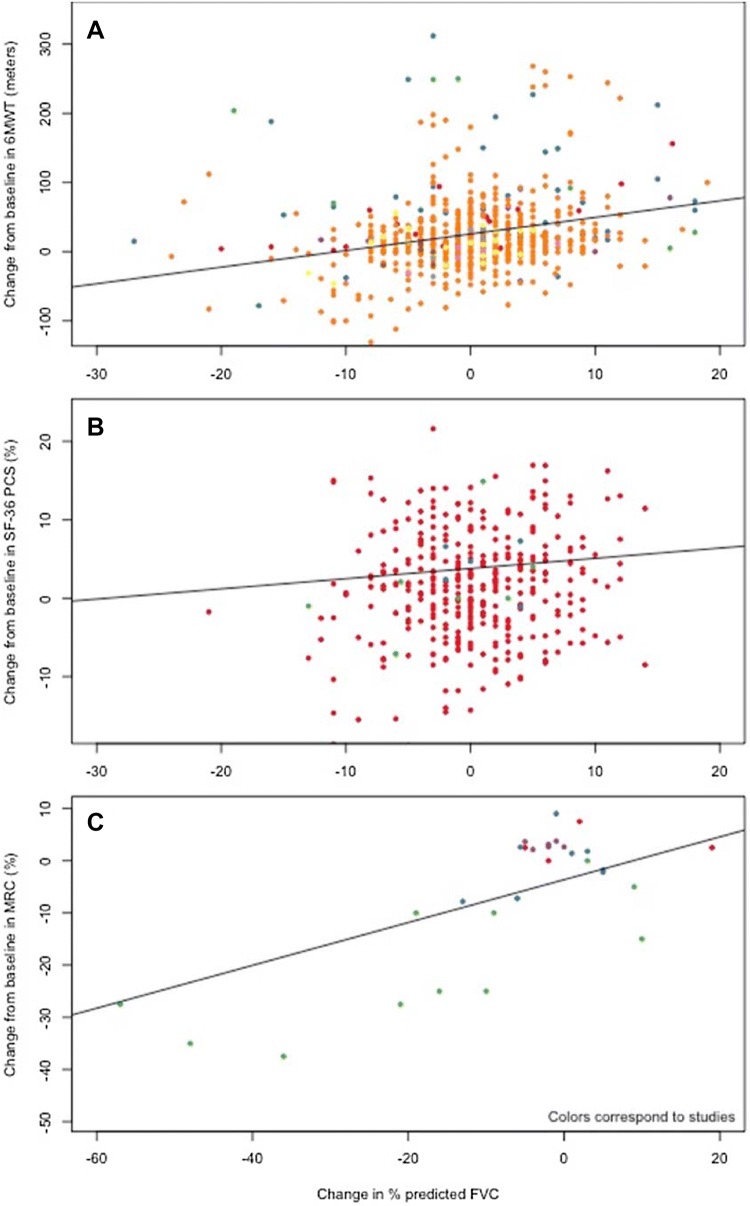
Association between change in percent predicted FVC and change in: **a** 6-min walk test; **b** SF-36 physical component score; and **c** MRC skeletal muscle strength score. Colors correspond to the studies making up the pooled cohort. In each case, the lines correspond to regression lines obtained through mixed-effects regression

Table 3 displays the results of mixed-effects linear models using longitudinal data to explore the association between changes in FVC and changes in each LOPD outcome. Figure 3 displays graphically the associations which were statistically significant. For most outcomes, the studies used were the same as those used in the cross-sectional analyses described above. For the 6MWT, the follow-up time of the ten included studies varied between 6 and 72 months. The relationship appeared to be positive and linear, with an interaction term for baseline values but, not as strong as in the cross-sectional analyses. The model adjusting for both baseline FVC and 6MWT was selected. From these, on average an improvement in FVC equal to 10% of predicted was associated with a 35.6-m (95% CI 19.91–51.57) improvement in the 6MWT for patients with an FVC of 55% at baseline; for patients with higher or lower values of FVC, the improvement in 6MWT was similar (34.63 m for FVC = 35% of predicted and 37.40 m for FVC = 85% of predicted).

**Table 3:** Modelled association between FVC% and LOPD outcomes using longitudinal analyses

Effect	Effect of FVC on LOPD outcome		Average change in LOPD outcome based on a 10% improvement in FVC (95% CI)
	Estimate (SE)	*p* value	
6-min walk test	3.27 (1.040)	0.0018	35.60 meters^a^ (19.91 to 51.57)
SF-36 PCS	0.13 (0.064)	0.0439	1.34% (0.08 to 2.60)
Maximal inspiratory pressure	0.14 (0.094)	0.0930	1.39 cmH_2_O (− 0.47 to 3.19)
Maximal expiratory pressure	0.06 (0.059)	0.3202	0.59 cmH_2_O (− 0.58 to 1.76)
Medical Research Council	0.41 (0.129)	0.0085	4.12% (1.29 to 6.95)

All models included an intercept and log time, except for the 6MWT, which also included baseline FVC, an interaction term between FVC and baseline FVC, as well as whether a patient had baseline 6MWT above 500 m. The other exception was MRC, which did not include time

*FVC* forced vital capacity, *SE* standard error, *CI* confidence interval

^a^Calculated at baseline FVC of 55%

For SF-36, the association between changes was more difficult to distinguish visually (Fig. 3b). Nonetheless, results of the hierarchical mixed-effects modelling (Table 3) suggest that the modelled relationships between changes in FVC and SF-36 PCS was positive and statistically significant. These results indicate that on average an increase in FVC equal to 10% of predicted was associated with an improvement of 1.3% (95% CI 0.05, 2.55) in SF-36 PCS score. There were no statistical associations between change in FVC and change in MIP/MEP (Table 3).

### Other outcomes

Five studies were included in the analysis of Walton and Gardner-Medwin scores for motor disability (WGM) [7, 11, 12, 14, 15, 19]. Results for this outcome are presented in the Web Appendix. No association was detected between FVC and GSGC scores. Finally, there were too few data for the following outcomes: RHS, FSS and survival.

## Discussion

The degree of pulmonary dysfunction is associated with morbidity across a wide range of diseases, i.e., individuals with lower values for pulmonary function metrics have more respiratory complaints and, in general, greater impairments in ability to work and function in daily life [23]. This is of particular importance in Pompe disease, where reduction in FVC is associated with an increased incidence of respiratory complications and death. In fact, studies in untreated Pompe patients have shown that the odds for use of ventilatory support increases by 8% for every year after diagnosis [2]. Any delay in the time to onset of ventilator support would have a meaningful impact on individual patient’s lives. This is especially true since the need for respiratory support marks a critical transition for patients and is associated with a significant reduction in self-reported quality of life as measured by the SF-36 [24] and the EuroQol-5 Dimension scale of quality of life (EQ-5D-5L) [25].

Previous systematic literature reviews and meta-analyses have investigated the effect of alglucosidase alfa on various measures assessing disease progression, with FVC being one of the central measures [25-27]. This study extends prior observations using a pooled cohort of LOPD patients to assess the association between FVC and other LOPD outcome measures, including assessments in non-respiratory domains. Moreover, this study performed both cross-sectional analyses, to assess the association between individuals at given FVC levels, and longitudinal analyses, to assess the association between changes in FVC and changes in other LOPD measures and outcomes. Within the cross-sectional analyses, there was strong evidence of positive associations between FVC and measures across a variety of domains including MIP and MEP among respiratory measures, the 6MWT among exercise measures, MRC among skeletal muscle strength measures, and SF-36 among patient-reported quality of life measures. These positive associations indicate that patients with preserved respiratory function also perform better in other domains, in turn suggesting that measures targeted to conserve FVC would help conserve other domains pertinent to LOPD. The meta-analyses by Schoser et al., 2016 demonstrated that alglucosidase alfa can be used to stabilize FVC [26].

The longitudinal analyses also demonstrated positive associations between changes in FVC and changes in other LOPD outcomes. These were strongest among the 6MWT and the MRC skeletal muscle strength scores. While MRC is a physiological variable, the 6MWT is a measure of functional endurance and the positive association with changes in FVC underscores the impact of respiratory function on exercise capacity. Overall, changes in FVC appear to be associated with changes in measures commonly used to assess disease severity in LOPD patients, and importantly these variables spanned respiratory, motor-skill and quality of life domains. Another interesting finding in this study was the demonstration of a larger change in 6MWT associated with changes in FVC at higher levels of baseline FVC in the longitudinal analysis. This observation highlights a potential benefit of initiating therapy while FVC is preserved at a higher level to maintain a higher functional status in individual patients.

The results of this study provide support for the notion that changes in FVC are correlated to changes in other health aspects related to LOPD. Given the heterogeneity of disease manifestations between individual patients, it was unclear whether associations would be found between respiratory and motor-skill variables. A strong association was seen between the observed values in the cross-sectional analysis, which aligns with observations seen in other neuromuscular diseases including amyotrophic lateral sclerosis and spinal cord injury [28, 29]. On the other hand, we found a weaker association between change in FVC and change in measures of respiratory muscle strength (i.e. MIP and MEP). With respect to MEP, this may be explained by the fact that MEP only accounts for ability to exhale below the resting lung volume, which numerically is only 25–33% of the FVC. With respect to MIP, this may be reflective of the fact that the relationship between FVC and MIP is known to be non-linear, as well as the difficulty of getting good respiratory muscle measurements in patients [30].

The pooled cohort used in the individual patient-level meta-analysis was developed to assess associations that could not be determined using traditional evidence synthesis methods. Relative to more commonly used forms of evidence synthesis, individual patient-level data meta-analyses are more robust and can be used to answer a wider range of research questions. While pairwise meta-analysis can be used to provide an estimate for a relative treatment effect, it cannot as easily be used to determine the relationship between multiple variables. Moreover, traditional meta-analytic methods can fall prey to the ecological fallacy, whereby a trend in summary statistics does not necessarily translate to a trend among individuals. Simulated cohorts tend to be underutilized because individual patient-level data are difficult to obtain; however, given that LOPD is a rare disease it was feasible to use this approach. Specifically, publications in this and other rare diseases are more likely to provide individual patient-level data. It is only through methods such as these that such large sample sizes can be obtained to draw insights into this rare disease area.

There have been previous systematic literature reviews and meta-analyses published in LOPD that have used summary statistics [26, 27]. Kanters and colleagues investigated a similar research question in which the objective was to investigate predictive models for a number of assessments within the cohort of LOPD patients seen at the Center for Lysosomal and Metabolic Diseases at Erasmus Medical Center from January 2005 to October 2009 [25]. The study found FVC to be a statistically significant predictor of the RHS. This complements our results given that there were insufficient data to analyze RHS within the pooled cohort.

The study has several strengths and limitations. Despite LOPD being a rare disease, the inclusion of the patient data from the LOTS trial provided a relatively large sample size and an even larger number of observations given the numerous repeated measures on each patient. Among the study limitations, the pooled cohort came from a variety of settings and designs, thus results are susceptible to selection bias. Although we did use a hierarchical approach to recognize the various clusters, limited and/or variable report of demographic and clinical variables mitigated the number of variables that could be used to adjust for potential selection bias. In particular, accounting for disease duration was identified a priori as a desirable model adjustment, but it was simply not feasible. Second, given that this is a rare condition, it is possible that one or more patients were included within multiple studies. Rigorous procedures were used to avoid this issue, and we are confident that nearly all measures were obtained in mutually exclusive patients (the only exception is the 6MWT, where the overlap would be limited to < 2.8% overlap). This potential bias is further minimized in analyses that include the LOTS trial given that they represent a large portion of the observations. Third, this study design did not conduct observations of long-term outcomes in the subgroup of patients with higher baseline FVC, which would be valuable for newly diagnosed patients that begin ERT with preserved respiratory function. Unfortunately, existing information is limited to clinical trial data and extension studies where the subject inclusion criteria required a variable degree of respiratory impairment at baseline. Finally, as to be expected with such a rare condition, the evidence base was sparse for outcomes not included in the LOTS trial. As a result, some analyses were underpowered rendering it difficult to determine statistical significance in observed trends. Nonetheless, statistically significant results were found among the analyses, which speaks to the strength of associations between respiratory outcomes and outcomes in other clinically important domains.

In summary, forced vital capacity is a respiratory outcome variable that is readily obtainable in the clinical setting and commonly reported in studies pertaining to LOPD patients. The evidence base analyzed and presented in this study demonstrates that FVC is positively associated with a variety of other LOPD measures and outcomes across multiple domains including endurance (6MWT), skeletal muscle strength (MRC) and patient-reported outcomes (SF-36). Moreover, longitudinal data indicate concordance between improvement in FVC and a corresponding improvement in other functional measures. These cross-sectional and longitudinal correlations are of particular importance since each of the LOPD outcome parameters reflects the function of different muscle groups. This study highlights the importance of assessing respiratory function to delineate treatment benefits in individuals with Pompe disease treated with enzyme replacement.

## Electronic supplementary material

Below is the link to the electronic supplementary material.

Supplementary file1 (DOCX 2102 kb)
