# Supplementary material for: Forced vital capacity and cross-domain late-onset Pompe disease outcomes: an individual patient-level data meta-analysis
Source: J Neurol. 2019 Jun 11;266(9):2312–21. doi: 10.1007/s00415-019-09401-1 (PMC6687674; doi:10.1007/s00415-019-09401-1)
Supplement: Supplementary file 1 — Supplementary file1 (DOCX 2102 kb) [file 415_2019_9401_MOESM1_ESM.docx]

**Web Appendix**

**Literature search strategies**

**Table 1: The search strategy employed for searches in Medline and Embase**

| **No.** | **Terms** | **Comments** |
| --- | --- | --- |
| 1. | exp Glycogen Storage Disease Type II/ | Population:  Late-onset Pompe Disease (LOPD) terms |
| 2. | (glycogen storage disease type II OR glycogen storage disease type 2 OR gsd?II OR gsd II OR gsd?2 OR gsd 2).mp. |  |
| 3. | (glycogenosis type II OR glycogenosis type 2).mp |  |
| 4. | (late* OR late-onset OR juvenile* OR adult* OR child* OR non-infantile*).mp. |  |
| 5. | (Pompe OR Pompe-disease OR lopd).mp. |  |
| 6. | (acid alpha-glucosidase deficiency OR gaa deficiency OR (deficiency AND (acid alpha-glucosidase OR gaa))).mp. |  |
| 7. | (acid maltase deficiency or AMD).mp. not (macular).mp. |  |
| 8. | 4 AND (5 OR 6 OR 7) |  |
| 9. | OR/1-3 OR 8 | Population results |
| 10. | exp enzyme replacement therapy/ | Intervention: Alglucosidase alfa terms |
| 11. | (enzyme replacement therapy OR ert OR enzyme therapy).mp. |  |
| 12. | (alglucosidase alfa OR alglucosidase* OR genzyme OR myozyme OR lumizyme OR rhGAA OR recombinant human GAA).mp. |  |
| 13. | OR/10-12 | Intervention results |
| 14. | (natural history OR natural course OR course*) | Comparison:  Natural history (prognosis) terms |
| 15. | exp mortality/ |  |
| 16. | incidence.sh. OR follow-up stud*.sh. |  |
| 17. | (prognos* OR predict*).mp. |  |
| 18. | OR/14-17 | Prognosis results |
| 19. | 8 AND (13 OR 18) | Final results |

mp denotes multi-purpose and implies a search through all fields; .sh. denotes a Medical Subject Heading (MeSH) term; exp denotes explode and implies that a term and a collection of variations on that term are searched for; * is used for truncation; ? is a single space wildcard term.

**Table 2: The search strategy employed to search Cochrane Central Register of Controlled Trials**

| **No.** | **Terms** | **Comments** |
| --- | --- | --- |
| 1. | exp Glycogen Storage Disease Type II/ | Population:  Late-onset Pompe Disease (LOPD) terms |
| 2. | (glycogen storage disease type II OR glycogen storage disease type 2 OR gsd?II OR gsd II OR gsd?2 OR gsd 2).mp. |  |
| 3. | (glycogenosis type II OR glycogenosis type 2).mp |  |
| 4. | (late* OR late-onset OR juvenile* OR adult* OR child* OR non-infantile*).mp. |  |
| 5. | (Pompe OR Pompe-disease OR lopd).mp. |  |
| 6. | (acid alpha-glucosidase deficiency OR gaa deficiency OR (deficiency AND (acid alpha-glucosidase OR gaa))).mp. |  |
| 7. | (acid maltase deficiency or AMD).mp. not (macular).mp. |  |
| 8. | 4 AND (5 OR 6 OR 7) |  |
| 9. | OR/1-3 OR 8 | Population results |
| 10. | exp enzyme replacement therapy/ | Intervention: Alglucosidase Alfa terms |
| 11. | (enzyme replacement therapy OR ert OR enzyme therapy).mp. |  |
| 12. | (alglucosidase alfa OR alglucosidase* OR genzyme OR myozyme OR lumizyme OR rhGAA OR recombinant human GAA).mp. |  |
| 13. | OR/10-12 | Intervention results |
| 14. | 8 AND 13 | Final results |

**Included and Excluded Studies**

**Table 3: List of included studies and publications reporting individual patient-level data**

| Trial ID | Author | Title | Year |
| --- | --- | --- | --- |
| Adreassen et al, 2014^43^ | Adreassen et al | Effect of enzyme replacement therapy on isokinetic strength for all major muscle groups in four patients with Pompe disease-a long-term follow-up | 2014 |
| Angelini et al, 2009^26^ | Angelini et al | Progress in enzyme replacement therapy in glycogen storage disease type ii | 2008 |
| Angelini et al, 2012^25^ | Angelini et al | Observational clinical study in juvenile-adult glycogenosis type 2 patients undergoing enzyme replacement therapy for up to 4 years | 2012 |
| Deroma et al 2014^15^ | Deroma et al | Enzyme replacement therapy in juvenile glycogenosis type II: a longitudinal study | 2014 |
| Gaeta et al 2013^16^ | Gaeta et al | Late-onset Pompe disease (LOPD): correlations between respiratory muscles CT and MRI features and pulmonary function | 2013 |
| Hundsberger et al 2014^17^ | Hundsberger et al | Cessation and resuming of alglucosidase alfa in Pompe disease: a retrospective analysis | 2014 |
| LOTS^4, 7^ | Van Der Ploeg et al | A randomized study of alglucosidase alfa in late-onset Pompe disease | 2010 |
|  | Van Der Ploeg et al | Open-label extension study following the Late-Onset Treatment Study (LOTS) of alglucosidase alfa | 2012 |
| Merk et al, 2009^27^ | Merk et al | Glycogen storage disease type ii (Pompe disease) - influence of enzyme replacement therapy in adults | 2009 |
| Montagnese et al 2015^18^ | Montagnese et al | Clinical and molecular aspects of 30 patients with late-onset Pompe disease (LOPD): unusual features and response to treatment | 2014 |
| Orlikowski et al, 2011^44^ | Orlikowski et al | Recombinant human acid alpha-glucosidase (rhgaa) in adult patients with severe respiratory failure due to Pompe disease | 2011 |
| Papadimas et al, 2011^29^ | Papadimas et al | Adult Pompe disease: Clinical manifestations and outcome of the first Greek patients receiving enzyme replacement therapy | 2011 |
| Patel et al, 2012^23^ | Patel et al | The impact of antibodies in late-onset Pompe disease: A case series and literature review | 2012 |
| Van Capelle et al, 2010^45^ | Van Capelle et al | Effect of enzyme therapy in juvenile patients with Pompe disease: A three-year open-label study | 2010 |
| Van Capelle et al, 2010b^46^ | Van Capelle et al | Eight years experience with enzyme replacement therapy in two children and one adult with Pompe disease | 2010 |
| Van Der Beek et al, 2009^47^ | Van Der Beek et al | Rate of disease progression during long-term follow-up of patients with late-onset Pompe disease | 2009 |
| Vielhaber et al 2011^20^ | Vielhaber et al | 24-months results in two adults with Pompe disease on enzyme replacement therapy | 2011 |

**Table 4: List of included studies and publications reporting summary statistics only**

| Trial ID | Author | Title | Year |
| --- | --- | --- | --- |
| Boentert M et al 2015^14^ | Boentert M et al | Sleep‐related symptoms and sleep‐disordered breathing in adult Pompe disease | 2015 |
| De Vries et al, 2012^48^ | De Vries et al | Effect of enzyme therapy and prognostic factors in 69 adults with Pompe disease: An open-label single-center study | 2012 |
| EMBASSY^19^ | Thurberg et al | A Phase 4 Prospective Study in Patients with Adult Pompe Disease Treated with Alglucosidase Alfa (S50.002) | 2015 |
| Gungor et al, 2013^33^ | Gungor et al | Impact of enzyme replacement therapy on survival in adults with Pompe disease: Results from a prospective international observational study | 2013 |
| LOPOS^49^ | Wokke et al | Clinical features of late-onset Pompe disease: A prospective cohort study | 2008 |
| NCS-LSD cohort study^31^ | Anderson et al | Effectiveness of enzyme replacement therapy in adults with late-onset Pompe disease: results from the NCS-LSD cohort study | 2014 |
| Restel et al, 2014^50^ | Restel et al | Enzymatic replacement therapy in patients with late onset Pompe disease-5-year follow up | 2014 |
| Strothotte/Regnery^35^ | Strothotte et al | Enzyme replacement therapy with alglucosidase alfa in 44 patients with late-onset glycogen storage disease type 2: 12-month results of an observational clinical trial | 2010 |
|  | Regnery et al | 36 months observational clinical study of 38 adult Pompe disease patients under alglucosidase alfa enzyme replacement therapy | 2012 |
| Van Der Beek et al, 2012^48^ | Van Der Beek et al | Clinical features and predictors for disease natural progression in adults with Pompe disease: A nationwide prospective observational study | 2012 |

**Table 5: List of studies excluded at full-text screening**

| **Author** | **Year** | **Title** | **Reason** |
| --- | --- | --- | --- |
| Alejaldre, A., et al.^51^ | 2012 | Trunk muscle involvement in late-onset Pompe disease: Study of thirty patients | Outcomes |
| An, Y., et al.^52^ | 2005 | Glucose tetrasaccharide as a biomarker for monitoring the therapeutic response to enzyme replacement therapy for Pompe disease | Outcomes |
| Angelini, C. and S. Prasad^53^ | 2011 | Pompe disease diagnosis, treatment, and outcomes in Italy: Pompe disease registry data from Italy compared with the rest-of-world | Outcomes |
| Angelini, C., et al.^54^ | 2011 | Long-term follow-up effects on enzyme replacement treatment of adult form of acid maltase deficiency myopathy | Outcomes |
| Angelini, C., et al.^55^ | 2011 | Enzyme replacement therapy (ERT) in glycogen storage disease type ii: The first treatment developed | Outcomes |
| Angelini, C., et al.^56^ | 2012 | New motor outcome function measures in evaluation of late-onset Pompe disease before and after enzyme replacement therapy | Study design |
| Angelini, C., et al.^57^ | 2010 | Role of autophagy and atrophy in different GSDII phenotypes: How a survival response becomes a pathogenetic mechanism | Outcomes |
| Angelini, C., et al.^26^ | 2009 | Progress in enzyme replacement therapy in glycogen storage disease type ii | Other |
| Annane, D. and D. Orlikowski^58^ | 2008 | Pompe disease in adults-advanced stage | Study design |
| Annane, D., et al.^59^ | 2010 | Clinical signs and symptoms of Pompe disease in 143 infantile-onset and 424 late-onset patients: A report from the Pompe registry | Other |
| Banos Alvarez, I. and P. A. Miranda Machado^60^ | 2014 | Enfermedad de Pompe de inicio tardio con compromiso respiratorio severo, influencia de la terapia de reemplazo enzimatico | Study design |
| Barca, E., et al.^61^ | 2013 | Clinical, morphological and genetic features of a cohort of late onset GSDII patients: Typical and atypical presentations | Outcomes |
| Bembi, B., et al.^62^ | 2009 | Enzyme replacement therapy with alglucosidase alfa in juvenile-adult glycogenosis type 2 patients | Other |
| Bembi et al^21^ | 2010 | Long-term observational, non-randomized study of enzyme replacement therapy in late-onset glycogenosis type ii | Outcomes and overlaps with other publications |
| Bereznai, B., et al.^63^ | 2011 | Clinical manifestation, disease course and response to enzyme replacement therapy in Hungarian patients with Pompe's disease. [Hungarian] | Other |
| Boentert, M., et al.^64^ | 2013 | Sleep quality and sleep-related symptoms in Pompe disease | Outcomes |
| Byrne, B. J., et al.^65^ | 2014 | Phase i/ii trial of diaphragm delivery of recombinant adeno-associated virus acid alpha-glucosidase (raaav1-cmv-gaa) gene vector in patients with Pompe disease | Interventions |
| Byrne, B. J., et al.^66^ | 2011 | Pompe disease: Design, methodology, and early findings from the Pompe registry | Study design |
| Capelle, C. V.^67^ | 2008 | Pompe disease in children-early to mid-stage | Study design |
| Chien, Y. H., et al.^68^ | 2013 | Myostatin and insulin-like growth factor i: Potential therapeutic biomarkers for Pompe disease | Outcomes |
| Cisilino, G., et al.^69^ | 2011 | Long term effects of enzyme replacement therapy in juvenile Pompe patients: A 48 months follow up study | Outcomes |
| Corzo, D., et al.^70^ | 2008 | Alglucosidase alfa (Myozyme) in infants and children with rapidly progressive Pompe disease | Population |
| De Filippi, P., et al.^71^ | 2010 | The angiotensin-converting enzyme insertion/deletion polymorphism modifies the clinical outcome in patients with Pompe disease | Outcomes |
| De Vries, J. M., et al.^72^ | 2011 | Enzyme replacement therapy and prognostic factors for response an ongoing open-label cohort study in adults with Pompe disease | Other |
| De Vries et al^73^ |  | Effects of antibody formation during enzyme replacement therapy in 73 adult patients with Pompe disease | Outcomes: Relationship to FVC is simply described |
| Doche et al^74^ | 2014 | Enzymotherapy in late onset Pompe disease patients: A 4-year longitudinal study using quantitative MRI | Outcomes |
| Fiumara, A.^75^ | 2014 | Enzyme replacement therapy (ERT) in Pompe disease | Study design |
| Furusawa et al^22^ | 2012 | Effects of enzyme replacement therapy on five patients with advanced late-onset glycogen storage disease type II: A 2-year follow-up study | Outcomes: Only reports FVC |
| Gaeta, M., et al.^38^ | 2013 | Late-onset Pompe disease (LOPD): Correlations between respiratory muscles CT and MRI features and pulmonary function | Outcomes |
| Garcia, P., et al.^76^ | 2011 | Muscular strength and function in late-onset Pompe disease: Five-year follow-up of patients receiving enzyme replacement therapy | Outcomes |
| Gungor, D., et al.^77^ | 2012 | Effect of enzyme replacement therapy on fatigue in adults with Pompe disease | Other |
| Gungor, D., et al.^78^ | 2013 | Enzyme replacement therapy and fatigue in adults with Pompe disease | Outcomes |
| Gungor, D., et al.^79^ | 2013 | Impact of enzyme replacement therapy on survival in adults with Pompe disease | Duplicate Publication |
| Gungor, D., et al.^80^ | 2011 | Survival and prognostic factors prior to enzyme replacement therapy in 302 children and adults with Pompe disease | Outcomes |
| Gungor, D., et al.^81^ | 2011 | Survival and associated factors in 268 adults with Pompe disease prior to treatment with enzyme replacement therapy | Population |
| Gungor, D., et al.^82^ | 2011 | Survival of adult Pompe patients with and without enzyme replacement therapy | Outcomes |
| Haaker, G., et al.^83^ | 2014 | Orthopedic management of patients with Pompe disease: A retrospective case series of 8 patients | Interventions |
| Hagemans, M. L. C., et al.^84^ | 2007 | Impact of late-onset Pompe disease on participation in daily life activities: Evaluation of the Rotterdam handicap scale | Outcomes |
| Hagemans, M. L. C., et al.^85^ | 2005 | Disease severity in children and adults with Pompe disease related to age and disease duration | Study design |
| Hagemans, M. L. C., et al.^86^ | 2005 | Clinical manifestation and natural course of late-onset Pompe's disease in 54 Dutch patients | Study design |
| Hagemans, M. L., et al.^86^ | 2004 | Late-onset Pompe disease primarily affects quality of life in physical health domains | Other |
| Hagemans et al^87^ | 2006 | Course of disability and respiratory function in untreated late-onset Pompe disease | Outcomes and overlaps with other publications |
| Hartung, R., et al.^88^ | 2007 | Initial therapy response of 6 months of enzyme replacement therapy in 11 juvenile/adult m. Pompe patients | Other |
| Herzog, A., et al.^89^ | 2011 | Pompe disease in 42 German patients: Natural course, biochemical and molecular analysis | Outcomes |
| Herzog et al^90^ | 2012 | Pompe disease in 42 German patients: Natural course, biochemical and molecular analysis | Outcomes: Only reports FVC |
| Hobson-Webb, L. D., et al.^91^ | 2011 | The clinical and electrodiagnostic characteristics of Pompe disease with post-enzyme replacement therapy findings | Outcomes |
| Illes, Z., et al.^92^ | 2014 | Motor function and respiratory capacity in patients with late-onset Pompe disease | Study design |
| Jawdat, O., et al.^93^ | 2014 | Late-onset Pompe disease: A case series | Outcomes |
| Jin, W. N., et al.^94^ | 2014 | Clinical study of respiratory function in patients with late-onset glycogen storage disease type ii. [Chinese] | Other |
| Kanters, T. A., et al.^95^ | 2011 | Burden of illness of Pompe disease in patients only receiving supportive care | Outcomes |
| Kishnani P, et al^96^ | 2009 | Early treatment with alglucosidase alpha prolongs long-term survival of infants with Pompe disease | Study design |
| Kishnani, P. S., et al.^97^ | 2013 | Timing of diagnosis of patients with Pompe disease: Data from the Pompe registry | Outcomes |
| Kishnani, P., et al.^98^ | 2011 | The Pompe registry: Baseline data from the first five years | Outcomes |
| Kishnani, P., et al.^99^ | 2011 | The heterogeneity of Pompe disease: Early data on genotype from the Pompe registry | Outcomes |
| Kishnani, P., et al.^100^ | 2009 | Clinical signs and symptoms of Pompe disease in 120 infantile-onset and 373 late-onset patients: A report from the Pompe registry | Other |
| Kroos, M. A., et al.^101^ | 2007 | Broad spectrum of Pompe disease in patients with the same c.-32-13t->g haplotype | Outcomes |
| Laforet, P., et al.^102^ | 2013 | The French Pompe registry. Baseline characteristics of a cohort of 126 patients with adult Pompe disease | Other |
| Laforet, P., et al.^103^ | 2011 | Results of prolonged follow-up of late-onset Pompe disease treated with alglucosidase alfa (Myozyme) | Outcomes |
| Lianou, D., et al.^104^ | 2013 | Pompe disease (PD): Clinical outcome in infants and children treated with recombinant human acid alpha-glucosidase (rhgaa) | Population |
| Lin, D. S., et al.^105^ | 2013 | Low-frequency enzyme replacement therapy in late-onset Pompe disease | Study design |
| Merlini, L., et al.^106^ | 2010 | An exploratory analysis of scoliosis in 182 children and adults with Pompe disease from the Pompe registry | Outcomes |
| Muller-Felber, W., et al.^107^ | 2007 | Late onset Pompe disease: Clinical and neurophysiological spectrum of 38 patients including long-term follow-up in 18 patients | Outcomes |
| Musumeci, O., et al.^108^ | 2013 | Clinical and genetic features in a cohort of late onset glycogen storage disease type ii (GSDII) patients: Typical and atypical presentations | Outcomes |
| Musumeci, O., et al.^109^ | 2012 | MRI evaluation of respiratory function in patients with the late-onset form of Pompe disease | Outcomes |
| Nascimbeni, A. C., et al.^110^ | 2012 | Impaired autophagy contributes to muscle atrophy in glycogen storage disease type ii patients | Outcomes |
| Nascimbeni, A. C., et al.^111^ | 2012 | The role of autophagy in the pathogenesis of glycogen storage disease type ii (GSDII) | Outcomes |
| Nicolino, M., et al.^112^ | 2009 | Clinical outcomes after long-term treatment with alglucosidase alfa in infants and children with advanced Pompe disease | Population |
| Papadimas, G. K., et al.^113^ | 2012 | Bone density in patients with late onset Pompe disease | Outcomes |
| Papadimas, G., et al.^114^ | 2009 | Three years experience with enzyme replacement therapy in Greek patients with adult Pompe's disease | Other |
| Patel, T., et al.^115^ | 2012 | Immunological challenges in late-onset Pompe disease treated with enzyme replacement therapy | Outcomes |
| Pellegrini, N., et al.^116^ | 2005 | Respiratory insufficiency and limb muscle weakness in adults with Pompe's disease | Outcomes |
| Pichiecchio, A., et al.^117^ | 2009 | Enzyme replacement therapy in adult-onset glycogenosis ii: Is quantitative muscle MRI helpful? | Outcomes |
| Ploeg, A. V. D., et al.^118^ | 2008 | Natural course and effects of enzyme therapy in adults with Pompe disease | Study design |
| Ravaglia, S., et al.^119^ | 2010 | Changes in nutritional status and body composition during enzyme replacement therapy in adult-onset type ii glycogenosis | Outcomes |
| Ravaglia, S., et al.^120^ | 2010 | Prognostic factors for late-onset Pompe disease with enzyme replacement therapy: The two sides of low BMI | Study design |
| Ravaglia, S., et al.^121^ | 2009 | Enzyme replacement therapy in late-onset type ii glycogenosis | Study design |
| Restel, M., et al.^50^ | 2014 | Encymatic replacement therapy in patients with late-onset Pompe's disease - a 5-year follow up | Duplicate Publication |
| Roberts, M., et al.^122^ | 2011 | The prevalence and impact of scoliosis in Pompe disease: Lessons learned from the Pompe registry | Study design |
| Rodrigues, F., et al.^123^ | 2011 | Psychological follow-up of late-onset Pompe patients and parents expectations during 4 years of enzyme replacement therapy | Outcomes |
| Rossi, M., et al.^124^ | 2007 | Long-term enzyme replacement therapy for Pompe disease with recombinant human alpha-glucosidase derived from Chinese hamster ovary cells | Outcomes |
| Sacconi, S., et al.^125^ | 2014 | Atrioventricular block requiring pacemaker in patients with late onset Pompe disease | Interventions |
| Saux, A., et al.^126^ | 2008 | A retrospective study of six patients with late-onset Pompe disease. [French] | Other |
| Schneider, I., et al.^127^ | 2013 | Respiratory function in late-onset Pompe disease patients receiving long-term enzyme replacement therapy for more than 48 months | Other |
| Schoser, B.^128^ | 2013 | Alglucosidase alfa: 5 years of experience in late-onset Pompe disease | Study design |
| Sechi, A., et al.^129^ | 2014 | Does enzyme replacement therapy induce an acute improvement in exercise-tolerance in late-onset Pompe patients? | Outcomes |
| Slonim, A. E., et al.^130^ | 2007 | Modification of the natural history of adult-onset acid maltase deficiency by nutrition and exercise therapy | Interventions |
| Smith, B. K., et al.^131^ | 2013 | Phase i/ii trial of adeno-associated virus-mediated alpha-glucosidase gene therapy to the diaphragm for chronic respiratory failure in Pompe disease: Initial safety and ventilatory outcomes | Interventions |
| Smith, B. K., et al.^132^ | 2013 | Acid alpha-glucosidase gene replacement therapy to the diaphragm in ventilator-dependent Pompe disease: One-year respiratory motor outcomes | Interventions |
| Terzis, G., et al.^133^ | 2012 | Effects of exercise training during infusion on late-onset Pompe disease patients receiving enzyme replacement therapy | Interventions |
| Terzis, G., et al.^134^ | 2011 | Effect of aerobic and resistance exercise training on late-onset Pompe disease patients receiving enzyme replacement therapy | Interventions |
| Tolun, A. A., et al.^135^ | 2011 | Monitoring urinary glucose tetrasaccharide biomarker in patients with infantile and late-onset Pompe disease identified through newborn screening | Outcomes |
| Van Den Berg, L. E. M., et al.^136^ | 2013 | Muscle fiber-type distribution, fiber-type-specific damage, and the Pompe disease phenotype | Outcomes |
| Van Den Berg, L., et al.^137^ | 2013 | Effects of exercise training in 23 adults with Pompe disease receiving enzyme therapy | Outcomes |
| Van Der Beek, N. a. M. E., et al.^138^ | 2011 | Rate of progression and predictive factors for pulmonary outcome in children and adults with Pompe disease | Duplicate Publication |
| Van Der Beek, N. a. M. E., et al.^139^ | 2010 | Rate of disease progression and response to enzyme replacement therapy in adults with Pompe disease | Other |
| Van Der Ploeg A, et al^140^ | 2008 | Placebo-controlled study of alglucosidase alfa in adults with Pompe disease [abstract] | Other |
| Van Der Ploeg, A. T., et al.^141^ | 2008 | Results from a randomized, double-blind, multicenter, multinational, placebo-controlled study of the safety and efficacy of Myozyme, recombinant human acid alpha-glucosidase (rhgaa), for the treatment of Pompe disease in juveniles and adults | Other |
| Vianello, A., et al.^142^ | 2013 | Enzyme replacement therapy improves respiratory outcomes in patients with late-onset type ii glycogenosis and high ventilator dependency | Other |
| Vielhaber, S., et al.^20^ | 2011 | 24-months results in two adults with Pompe disease on enzyme replacement therapy | Study design |
| Wens et al, 2015^143^ | 2015 | Lung MRI and impairment of diaphragmatic function in Pompe disease | Population |
| Wilson, A. B., et al.^144^ | 2011 | Selecting an appropriate comparison group: Characterizing treated and untreated patients in the Pompe registry | Outcomes |
| Winkel, L. P. F., et al.^145^ | 2004 | Enzyme replacement therapy in late-onset Pompe's disease: A three-year follow-up | Interventions |
| Wirsching, A., et al.^146^ | 2014 | Are evoked potentials in patients with adult-onset Pompe disease indicative of clinically relevant central nervous system involvement? | Outcomes |
| Wu, K. H. C., et al.^147^ | 2012 | An observational study of nine adult late-onset Pompe disease patients treated or untreated with acid alpha-glucosidase enzyme replacement therapy | Outcomes |
| Wyatt, K., et al.^148^ | 2012 | The effectiveness and cost-effectiveness of enzyme and substrate replacement therapies: A longitudinal cohort study of people with lysosomal storage disorders | Outcomes |
| Yang, C. C., et al.^149^ | 2011 | Rapid progressive course of later-onset Pompe disease in Chinese patients | Outcomes |
| Yang et al^149^ | 2011 | Rapid progressive course of later-onset Pompe disease in Chinese patients | Outcomes |
| Yonee, C., et al.^150^ | 2012 | Quantitative computed tomography for enzyme replacement therapy in Pompe disease | Outcomes |
| Young, S. P., et al.^151^ | 2012 | Assessing disease severity in Pompe disease: The roles of a urinary glucose tetrasaccharide biomarker and imaging techniques | Outcomes |
| Zagnoli, F.^152^ | 2013 | Late-onset Pompe disease: Modest effect for the replacement for ERT, but a first treatment in myology. [French] | Study design |

**These studies are those that were considered in full text for this project, including papers that were included in the previous review.*

**Methods – Additional details**

LOPD is an ultra-orphan disease and as such there was a risk of patient overlap between studies. We used multiple criteria to identify patient overlap, with geography playing a primary role. Studies that were the only to report within a particular country were deemed to not have patient overlap. Studies from the same country within the same centers and universities were deemed to overlap in patients (conditional on overlapping timelines). Other studies discussed the overlap explicitly within the papers. Finally, if there was still a lack of clarity regarding patient overlap following these steps, individual demographics from the IPD were investigated to identify potential repeats. *A priori*, we judged that a 10% overlap in studies reporting summary statistics was negligible. Overlap in and of itself was not grounds for exclusion unless a smaller study offered no novel outcomes.

The quality of RCTs was assessed using the Risk of Bias instrument,^10^ endorsed by the Cochrane Collaboration. An additional tool that was used to assess methodological study quality was the Downs and Black instrument, which is applicable to both randomized and non-randomized studies.^11^ Assessments of study quality were independently performed by two reviewers (AB and GS) trained in using these scoring instruments. Disagreements arising between reviewers were resolved by a third adjudicator. The results of the quality assessment are provided in the next section.

Given the nature of the research question, the basic model involved a selected outcome with FVC as a predictor. Change from baseline values were calculated if only observed values were presented and vice versa. The outcome variables were: 6MWT, MRC, RHS, FSS, SF-36 PCS, WGM, GSGC, MIP, MEP, and Survival. FVC data were primarily reported in percentages, representing the proportion between observed FVC and expected FVC. Expected FVC is calculated using age, sex and height. Measurements reported in liters were converted to percentage using the equations for calculating expected FVC.^8^ Specifically, the equation for men is *Expected FVC = 5.76 x height - 0.026 x age - 4.34* and the equation for women is *Expected FVC = 4.43 x height - 0.026 x age - 2.89*. Individual ages were used when available (most often they were available) and average age used otherwise. Average height for the study locations (Denmark and Italy)^9^ were considered in the conversion process, as no height information was available in those papers.

The second analytical stage involved using statistical models to determine the association between changes in FVC and changes in LOPD outcomes. To account for the clustered nature of the synthetic cohort, nested hierarchical modeling was used. Specifically, we used generalized linear and non-linear mixed models.^12^ For all the models fit in these analyses, combinations of change FVC, baseline FVC, time, treatment with Myozyme and interactions between them were used as fixed-effects. For the 6MWT, we also used a dichotomous variable for having a baseline value of 500m or more as such patients were deemed to be unlikely to improve based on their starting point. Individual patients and studies were used as random-effects. Random effects can be used to construct random intercepts and random slopes. For these analyses, we chose to use a random-intercept approach.

The data used to conduct these analyses were unbalanced in nature. Cluster sizes varied both across and within studies. With respect to studies as a clustering variable, all studies had multiple measurements; however, with respect to time as a clustering variable, not all clusters had repeat measures. Nonetheless, in most analyses the LOTS trial represented a large proportion of the data and included multiple time measurements for all participants. As a result, there were relatively few patients with single observations. Clusters having just one observation contribute influence to both the estimated variance of the random-effect and the slope of the fixed-effect. This is because the random intercept is never actually estimated. As such it is completely feasible to conduct generalized mixed models with this form of unbalanced data. Numerical instability may happen, particularly in situations with many clusters with no repeated measures, but there was no evidence of such issues in the analyses we conducted.

Nonetheless, there was one modeling approach that did lead to numerical issues. Clustering can be approached using mixed effects or through marginal models, referred to as generalized estimating equations.^12^ We did attempt to use a random-effect for the studies and covariance structure for time, but this led to numerical issues pertaining to non-invertible matrices. The advantage of this approach would have been the ability to use auto-regressive correlation for measures repeated over time, but it is unlikely that this would have changed the model in any meaningful way.

**Figure 1: Clustering within the synthetic cohort of LOPD patients**


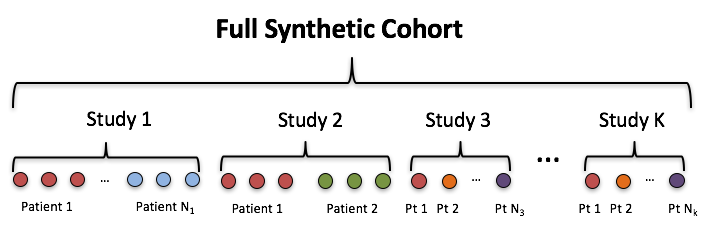


**Study characteristics**

**Table 6: Study and patient characteristics for trials reporting individual patient-level data**

| **Primary Author (Year)** | **Treatment** | **Sample**  **Size** | **Study**  **Design** | **Study Duration  (Months)** | **Region** | **Male (%)** | **Age at study onset (Years)** | **Disease Duration (Years)** | **Proportion in wheelchairs (%)** | **Proportion requiring ventilators (%)** | **FVC (Upright/**  **Sitting position)** |
| --- | --- | --- | --- | --- | --- | --- | --- | --- | --- | --- | --- |
| Adreassen et al, 2014^43^ | Myozyme | 4 | Cohort | 72 | Denmark | 3 (75) | 48 (10.03) | 2-11* | 0 (0) | 1 (25) | -- |
| Angelini et al, 2009^26^ | Myozyme | 11 | Cohort | 18 | Italy | 3 (27.2) | -- | 14.4 (7.7) | 2 (18) | 1 (9.1) | 72.2 (28.8) |
| Angelini et al, 2012^25^ | Myozyme | 74 | Cohort | 36 | Italy | 33 (44.5) | 43 (15.4) | 14.7 (8.7) | 7 (9.4) | 27 (36.4) | 65.2 (26.5) |
| Deroma et al 2014^15^ | Myozyme | 8 | Cohort | 72 | Italy | 5 (62.5) | 11 (3) | -- | 1 (12.5) | 2 (25) | 78.8 (41.1) |
| Gaeta et al 2013^16^ | No treatment | 10 | Cross-sectional | -- | Italy | 5 (50) | 42.7 (18.25) | 10.2 (4.3) | -- | 3 (30) | 75.3 (21) |
| Hundsberger et al 2014^17^ | Myozyme | 7 | Cohort | -- | Switzerland | 3 (42.9) | 43 (--) | -- | 1 (14.3) | 2 (28.6) | 81.8 (14.6) |
| LOTS^4, 7^ | Myozyme | 60 | RCT | 19.5 | USA & Europe | 34 (57) | -- | 9 (6.3) | 0 (0) | 20 (33.3) | 55.4 (14.4) |
|  | No Treatment (Placebo) | 30 | RCT | 19.5 | USA & Europe | 11 (37) | -- | 10.1 (8.4) | 0 (0) | 11 (36.6) | 53 (15.7) |
| Merk et al, 2009^27^ | Myozyme | 4 | Cohort | 6 | Germany | 0 (0) | -- | -- | 1 (25) | 2 (50) | -- |
| Montagnese et al 2015^18^ | Mixed | 30 | Cohort | 60 | Italy | 15 (50) | 47 (18) | -- | -- | -- | 71.7 (16.1) |
| Orlikowski et al, 2011^153^ | Myozyme | 5 | Cohort | 12 | France | 2 (40) | 47.8 (14.39) | 23.4 (6.58) | 3 (60) | 5 (100) | -- |
| Papadimas et al, 2011^29^ | Myozyme | 5 | Cohort | 38 | Greece | 1 (20) | 48.6 (13.93) | -- | 1 (20) | 1 (20) | -- |
| Patel et al, 2012^23^ | Myozyme | 3 | Case series | 52 | USA & Germany | 1 (33.3) | -- | -- | 0 (0) | 0 (0) | 87.3 (22.2) |
| Van Capelle et al, 2010^45^ | Myozyme | 5 | Cohort | 36 | Netherlands | 3 (60) | -- | -- | 0 (0) | 1 (20) | 81.54 (18.33) |
| Van Capelle et al, 2010b^46^ | Myozyme | 2 | Case series | 60 | Netherlands | 2 (66.6) | 19.7 (10.9) | 10 (12.1) | 2 (66.6) | 2 (66.6) | -- |
| Van Der Beek et al, 2009^47^ | No Treatment | 16 | Cohort | 192 | Netherlands | 6 (37.5) | -- | -- | 0 (0) | 4 (25) | -- |
| Vielhaber et al 2011^20^ | Myozyme | 2 | Case series | 24 | Germany | 1 (50) | 41.5 (0.5) | 11.5 (1.5) | 0 (0) | 0 (0) | 87.5 (9.2) |

*All data are mean (sd) unless specified; * Data are range; †Data are median (range); ** Data are mean; ††Data are median (IQR); --: Not reported FVC: forced vital capacity.*

**Reported patient level data**

These tables only present publicly available data (i.e., they do not include IPD for the LOTS trial)

Table 7. Individual patient level data for observed values, and change from baseline in: forced vital capacity, six-minute walking test, SF-36 physical component score, maximal inspiratory pressure, and maximal expiratory pressure

| **Study & Patient ID** | **Treatment** | **Time**  **(months)** | **Age** | **Gender** | **Disease duration**  **(years)** | **Age at diagnosis**  **(years)** | **Baseline FVC** | **Baseline 6MWT** | **FVC obs.** | **Change in FVC** | **6MWT obs.** | **Change in 6MWT** | **SF-36 obs.** | **Change in SF-36** | **MIP obs.** | **Change in MIP** | **MEP obs.** | **Change in MEP** |
| --- | --- | --- | --- | --- | --- | --- | --- | --- | --- | --- | --- | --- | --- | --- | --- | --- | --- | --- |
| Angelini et al, 2009 – 1 | Myozyme | 0 | 43 | Female | 8 | 54 | -- | -- | 80 | -- | 242 | -- | -- | -- | -- | -- | -- | -- |
| Angelini et al, 2009 – 10 | Myozyme | 12 | 43 | Female | -- | 22 | -- | -- | 99.6 | -- | 121 | -- | -- | -- | -- | -- | -- | -- |
| Angelini et al, 2009 – 10 | Myozyme | 6 | 43 | Female | -- | 22 | -- | -- | 88.4 | -- | 123 | -- | -- | -- | -- | -- | -- | -- |
| Angelini et al, 2009 – 11 | Myozyme | 0 | 43 | Female | 15 | 30 | -- | -- | 79.4 | -- | 125 | -- | -- | -- | -- | -- | -- | -- |
| Angelini et al, 2009 – 11 | Myozyme | 12 | 43 | Female | 15 | 30 | 90.6 | 501 | 83.4 | -5.9 | 128 | 40 | -- | -- | -- | -- | -- | -- |
| Angelini et al, 2009 – 11 | Myozyme | 18 | 43 | Female | 15 | 30 | 90.6 | 501 | 88.7 | -8.1 | 128 | 60 | -- | -- | -- | -- | -- | -- |
| Angelini et al, 2009 – 11 | Myozyme | 3 | 43 | Female | 15 | 30 | 90.6 | 501 | 81.9 | -2.2 | 209 | 8 | -- | -- | -- | -- | -- | -- |
| Angelini et al, 2009 – 11 | Myozyme | 6 | 43 | Female | 15 | 30 | 90.6 | 501 | 58.8 | -4.4 | 69 | 25 | -- | -- | -- | -- | -- | -- |
| Angelini et al, 2009 – 2 | Myozyme | 0 | 43 | Female | 22 | 15 | -- | -- | 62.6 | -- | 130 | -- | -- | -- | -- | -- | -- | -- |
| Angelini et al, 2009 – 2 | Myozyme | 12 | 43 | Female | 22 | 15 | 99.6 | 121 | 56.3 | -16 | 163 | 7 | -- | -- | -- | -- | -- | -- |
| Angelini et al, 2009 – 2 | Myozyme | 18 | 43 | Female | 22 | 15 | 99.6 | 121 | 13.4 | -10 | 125 | 7 | -- | -- | -- | -- | -- | -- |
| Angelini et al, 2009 – 2 | Myozyme | 3 | 43 | Female | 22 | 15 | 99.6 | 121 | 14.7 | -11 | 175 | 2 | -- | -- | -- | -- | -- | -- |
| Angelini et al, 2009 – 2 | Myozyme | 6 | 43 | Female | 22 | 15 | 99.6 | 121 | 14.9 | -20 | 169 | 4 | -- | -- | -- | -- | -- | -- |
| Angelini et al, 2009 – 4 | Myozyme | 0 | 43 | Male | 10 | 38 | -- | -- | 15.8 | -- | 130 | -- | -- | -- | -- | -- | -- | -- |
| Angelini et al, 2009 – 6 | Myozyme | 0 | 43 | Female | 24 | 45 | -- | -- | 81.9 | -- | 241 | -- | -- | -- | -- | -- | -- | -- |
| Angelini et al, 2009 – 6 | Myozyme | 3 | 43 | Female | 24 | 45 | 58.8 | 69 | 79.4 | 3.8 | 370 | 61 | -- | -- | -- | -- | -- | -- |
| Angelini et al, 2009 – 6 | Myozyme | 6 | 43 | Female | 24 | 45 | 58.8 | 69 | 83.4 | -2.5 | 397 | 94 | -- | -- | -- | -- | -- | -- |
| Angelini et al, 2009 – 7 | Myozyme | 0 | 43 | Female | 28 | 49 | -- | -- | 88.1 | -- | 429 | -- | -- | -- | -- | -- | -- | -- |
| Angelini et al, 2009 – 7 | Myozyme | 12 | 43 | Female | 28 | 49 | 13.4 | 125 | 91.5 | 2.4 | 468 | 5 | -- | -- | -- | -- | -- | -- |
| Angelini et al, 2009 – 7 | Myozyme | 3 | 43 | Female | 28 | 49 | 13.4 | 125 | 95.6 | 1.3 | 526 | 50 | -- | -- | -- | -- | -- | -- |
| Angelini et al, 2009 – 7 | Myozyme | 6 | 43 | Female | 28 | 49 | 13.4 | 125 | 97.4 | 1.5 | 448 | 44 | -- | -- | -- | -- | -- | -- |
| Angelini et al, 2009 – 8 | Myozyme | 0 | 43 | Female | 10 | 42 | -- | -- | 96.8 | -- | 462 | -- | -- | -- | -- | -- | -- | -- |
| Angelini et al, 2009 – 9 | Myozyme | 0 | 43 | Female | 9 | 39 | -- | -- | 90.6 | -- | 501 | -- | -- | -- | -- | -- | -- | -- |
| Angelini et al, 2009 – 9 | Myozyme | 12 | 43 | Female | 9 | 39 | 79.4 | 370 | 88.4 | 12.1 | 509 | 98 | -- | -- | -- | -- | -- | -- |
| Angelini et al, 2009 – 9 | Myozyme | 18 | 43 | Female | 9 | 39 | 79.4 | 370 | 86.2 | 16.2 | 526 | 156 | -- | -- | -- | -- | -- | -- |
| Angelini et al, 2009 – 9 | Myozyme | 3 | 43 | Female | 9 | 39 | 79.4 | 370 | 84.7 | 4 | 541 | 27 | -- | -- | -- | -- | -- | -- |
| Angelini et al, 2009 – 9 | Myozyme | 6 | 43 | Female | 9 | 39 | 79.4 | 370 | 82.5 | 8.7 | 561 | 59 | -- | -- | -- | -- | -- | -- |
| Angelini et al, 2012 – 10 | Myozyme | 0 | 72 | Male | -- | 53 | -- | -- | 85 | -- | 368 | -- | -- | -- | -- | -- | -- | -- |
| Angelini et al, 2012 – 10 | Myozyme | 12 | 72 | Male | -- | 53 | 80 | 340 | 89 | -33 | 460 | 44 | -- | -- | -- | -- | -- | -- |
| Angelini et al, 2012 – 11 | Myozyme | 0 | 41 | Female | -- | 28 | -- | -- | 69 | -- | 390 | -- | -- | -- | -- | -- | -- | -- |
| Angelini et al, 2012 – 11 | Myozyme | 48 | 41 | Female | -- | 28 | 70 | 197 | 73 | 4 | 410 | 90 | -- | -- | -- | -- | -- | -- |
| Angelini et al, 2012 – 12 | Myozyme | 0 | 39 | Female | -- | 38 | -- | -- | 95 | -- | 200 | -- | -- | -- | -- | -- | -- | -- |
| Angelini et al, 2012 – 12 | Myozyme | 12 | 39 | Female | -- | 38 | 62 | 360 | 82 | 1 | 439 | 150 | -- | -- | -- | -- | -- | -- |
| Angelini et al, 2012 – 13 | Myozyme | 0 | 68 | Female | -- | 44 | -- | -- | 79 | -- | 484 | -- | -- | -- | -- | -- | -- | -- |
| Angelini et al, 2012 – 13 | Myozyme | 24 | 68 | Female | -- | 44 | 68 | 140 | 53 | 10 | 370 | 20 | -- | -- | -- | -- | -- | -- |
| Angelini et al, 2012 – 15 | Myozyme | 0 | 37 | Female | -- | 16 | -- | -- | 56 | -- | 451 | -- | -- | -- | -- | -- | -- | -- |
| Angelini et al, 2012 – 16 | Myozyme | 0 | 66 | Female | -- | 42 | -- | -- | 80 | -- | 340 | -- | -- | -- | -- | -- | -- | -- |
| Angelini et al, 2012 – 16 | Myozyme | 24 | 66 | Female | -- | 42 | 65 | 71 | 47 | -6 | 384 | 79 | -- | -- | -- | -- | -- | -- |
| Angelini et al, 2012 – 17 | Myozyme | 0 | 28 | Female | -- | 7 | -- | -- | 70 | -- | 197 | -- | -- | -- | -- | -- | -- | -- |
| Angelini et al, 2012 – 18 | Myozyme | 0 | 43 | Female | -- | 40 | -- | -- | 74 | -- | 287 | -- | -- | -- | -- | -- | -- | -- |
| Angelini et al, 2012 – 18 | Myozyme | 12 | 43 | Female | -- | 40 | 56 | 194 | 62 | 9 | 360 | 71 | -- | -- | -- | -- | -- | -- |
| Angelini et al, 2012 – 19 | Myozyme | 0 | 37 | Male | -- | 20 | -- | -- | 63 | -- | 510 | -- | -- | -- | -- | -- | -- | -- |
| Angelini et al, 2012 – 2 | Myozyme | 0 | 55 | Female | -- | 44 | -- | -- | 68 | -- | 140 | -- | -- | -- | -- | -- | -- | -- |
| Angelini et al, 2012 – 2 | Myozyme | 36 | 55 | Female | -- | 44 | 85 | 368 | 78 | 4 | 160 | 92 | -- | -- | -- | -- | -- | -- |
| Angelini et al, 2012 – 21 | Myozyme | 0 | 57 | Female | -- | 28 | -- | -- | 103 | -- | 345 | -- | -- | -- | -- | -- | -- | -- |
| Angelini et al, 2012 – 22 | Myozyme | 0 | 36 | Male | -- | 27 | -- | -- | 65 | -- | 71 | -- | -- | -- | -- | -- | -- | -- |
| Angelini et al, 2012 – 23 | Myozyme | 0 | 61 | Female | -- | 33 | -- | -- | 59 | -- | 150 | -- | -- | -- | -- | -- | -- | -- |
| Angelini et al, 2012 – 24 | Myozyme | 0 | 46 | Female | -- | 29 | -- | -- | 79 | -- | 90 | -- | -- | -- | -- | -- | -- | -- |
| Angelini et al, 2012 – 24 | Myozyme | 24 | 46 | Female | -- | 29 | 110 | 346 | 56 | 0 | 194 | 54 | -- | -- | -- | -- | -- | -- |
| Angelini et al, 2012 – 25 | Myozyme | 0 | 47 | Male | -- | 42 | -- | -- | 65 | -- | 265 | -- | -- | -- | -- | -- | -- | -- |
| Angelini et al, 2012 – 25 | Myozyme | 12 | 47 | Male | -- | 42 | 64 | 435 | 19 | -8 | 50 | 22 | -- | -- | -- | -- | -- | -- |
| Angelini et al, 2012 – 26 | Myozyme | 0 | 60 | Female | -- | 44 | -- | -- | 51 | -- | 28 | -- | -- | -- | -- | -- | -- | -- |
| Angelini et al, 2012 – 26 | Myozyme | 48 | 60 | Female | -- | 44 | 53 | 256 | 72 | -2 | 592 | 56 | -- | -- | -- | -- | -- | -- |
| Angelini et al, 2012 – 27 | Myozyme | 0 | 18 | Male | -- | 3 | -- | -- | 22 | -- | 125 | -- | -- | -- | -- | -- | -- | -- |
| Angelini et al, 2012 – 27 | Myozyme | 12 | 18 | Male | -- | 3 | 109 | 511 | 110 | 1 | 346 | 61 | -- | -- | -- | -- | -- | -- |
| Angelini et al, 2012 – 28 | Myozyme | 0 | 29 | Male | -- | 20 | -- | -- | 110 | -- | 400 | -- | -- | -- | -- | -- | -- | -- |
| Angelini et al, 2012 – 28 | Myozyme | 36 | 29 | Male | -- | 20 | 89 | 495 | 64 | -10 | 435 | -38 | -- | -- | -- | -- | -- | -- |
| Angelini et al, 2012 – 29 | Myozyme | 0 | 40 | Male | -- | 36 | -- | -- | 56 | -- | 457 | -- | -- | -- | -- | -- | -- | -- |
| Angelini et al, 2012 – 29 | Myozyme | 12 | 40 | Male | -- | 36 | 78 | 448 | 53 | 7 | 256 | 149 | -- | -- | -- | -- | -- | -- |
| Angelini et al, 2012 – 30 | Myozyme | 0 | 38 | Female | -- | 14 | -- | -- | 51 | -- | 312 | -- | -- | -- | -- | -- | -- | -- |
| Angelini et al, 2012 – 30 | Myozyme | 36 | 38 | Female | -- | 14 | 73 | 504 | 109 | 7 | 511 | -36 | -- | -- | -- | -- | -- | -- |
| Angelini et al, 2012 – 31 | Myozyme | 0 | 52 | Male | -- | 24 | -- | -- | 110 | -- | 572 | -- | -- | -- | -- | -- | -- | -- |
| Angelini et al, 2012 – 31 | Myozyme | 36 | 52 | Male | -- | 24 | 22 | 75 | 89 | 6 | 495 | 42 | -- | -- | -- | -- | -- | -- |
| Angelini et al, 2012 – 32 | Myozyme | 0 | 34 | Female | -- | 20 | -- | -- | 79 | -- | 457 | -- | -- | -- | -- | -- | -- | -- |
| Angelini et al, 2012 – 32 | Myozyme | 12 | 34 | Female | -- | 20 | 65 | 394 | 78 | 9 | 448 | 25 | -- | -- | -- | -- | -- | -- |
| Angelini et al, 2012 – 33 | Myozyme | 0 | 12 | Male | -- | 2 | -- | -- | 85 | -- | 597 | -- | -- | -- | -- | -- | -- | -- |
| Angelini et al, 2012 – 33 | Myozyme | 36 | 12 | Male | -- | 2 | 138 | 617 | 73 | -16 | 504 | 188 | -- | -- | -- | -- | -- | -- |
| Angelini et al, 2012 – 34 | Myozyme | 0 | 59 | Male | -- | 30 | -- | -- | 80 | -- | 468 | -- | -- | -- | -- | -- | -- | -- |
| Angelini et al, 2012 – 35 | Myozyme | 0 | 40 | Male | -- | 37 | -- | -- | 22 | -- | 75 | -- | -- | -- | -- | -- | -- | -- |
| Angelini et al, 2012 – 35 | Myozyme | 36 | 40 | Male | -- | 37 | 80 | 360 | 28 | -4 | 117 | 60 | -- | -- | -- | -- | -- | -- |
| Angelini et al, 2012 – 37 | Myozyme | 0 | 48 | Male | -- | 30 | -- | -- | 65 | -- | 394 | -- | -- | -- | -- | -- | -- | -- |
| Angelini et al, 2012 – 37 | Myozyme | 36 | 48 | Male | -- | 30 | 89 | 210 | 74 | 15 | 419 | 212 | -- | -- | -- | -- | -- | -- |
| Angelini et al, 2012 – 38 | Myozyme | 0 | 41 | Male | -- | 26 | -- | -- | 138 | -- | 617 | -- | -- | -- | -- | -- | -- | -- |
| Angelini et al, 2012 – 39 | Myozyme | 0 | 41 | Male | -- | 26 | -- | -- | 122 | -- | 805 | -- | -- | -- | -- | -- | -- | -- |
| Angelini et al, 2012 – 4 | Myozyme | 0 | 45 | Male | -- | 34 | -- | -- | 54 | -- | 358 | -- | -- | -- | -- | -- | -- | -- |
| Angelini et al, 2012 – 4 | Myozyme | 18 | 45 | Male | -- | 34 | 69 | 390 | 80 | 4 | 360 | 20 | -- | -- | -- | -- | -- | -- |
| Angelini et al, 2012 – 40 | Myozyme | 0 | 51 | Female | -- | 42 | -- | -- | 76 | -- | 420 | -- | -- | -- | -- | -- | -- | -- |
| Angelini et al, 2012 – 40 | Myozyme | 24 | 51 | Female | -- | 42 | 84 | 287 | 89 | -3 | 210 | 94 | -- | -- | -- | -- | -- | -- |
| Angelini et al, 2012 – 41 | Myozyme | 0 | 7 | Female | -- | 2 | -- | -- | 104 | -- | 422 | -- | -- | -- | -- | -- | -- | -- |
| Angelini et al, 2012 – 41 | Myozyme | 36 | 7 | Female | -- | 2 | 93 | 573 | 61 | 11 | 192 | 29 | -- | -- | -- | -- | -- | -- |
| Angelini et al, 2012 – 42 | Myozyme | 0 | 37 | Female | -- | 15 | -- | -- | 16 | -- | 22 | -- | -- | -- | -- | -- | -- | -- |
| Angelini et al, 2012 – 42 | Myozyme | 48 | 37 | Female | -- | 15 | 89 | 77 | 84 | 2 | 287 | 195 | -- | -- | -- | -- | -- | -- |
| Angelini et al, 2012 – 43 | Myozyme | 0 | 65 | Male | -- | 55 | -- | -- | 81 | -- | 381 | -- | -- | -- | -- | -- | -- | -- |
| Angelini et al, 2012 – 43 | Myozyme | 24 | 65 | Male | -- | 55 | 67 | 378 | 93 | 18 | 573 | 73 | -- | -- | -- | -- | -- | -- |
| Angelini et al, 2012 – 46 | Myozyme | 0 | 12 | Male | -- | 2 | -- | -- | 104 | -- | 602 | -- | -- | -- | -- | -- | -- | -- |
| Angelini et al, 2012 – 46 | Myozyme | 36 | 12 | Male | -- | 2 | 27 | 104 | 89 | -5 | 77 | 249 | -- | -- | -- | -- | -- | -- |
| Angelini et al, 2012 – 47 | Myozyme | 0 | 18 | Male | -- | 17 | -- | -- | 91 | -- | 272 | -- | -- | -- | -- | -- | -- | -- |
| Angelini et al, 2012 – 47 | Myozyme | 18 | 18 | Male | -- | 17 | 78 | 510 | 67 | 7 | 378 | 40 | -- | -- | -- | -- | -- | -- |
| Angelini et al, 2012 – 49 | Myozyme | 0 | 56 | Male | -- | 30 | -- | -- | 85 | -- | 451 | -- | -- | -- | -- | -- | -- | -- |
| Angelini et al, 2012 – 49 | Myozyme | 36 | 56 | Male | -- | 30 | 28 | 75 | 27 | 4 | 104 | -42.5 | -- | -- | -- | -- | -- | -- |
| Angelini et al, 2012 – 5 | Myozyme | 0 | 48 | Female | -- | 25 | -- | -- | 22 | -- | 353 | -- | -- | -- | -- | -- | -- | -- |
| Angelini et al, 2012 – 50 | Myozyme | 0 | 13 | Male | -- | 4 | -- | -- | 78 | -- | 510 | -- | -- | -- | -- | -- | -- | -- |
| Angelini et al, 2012 – 50 | Myozyme | 36 | 13 | Male | -- | 4 | 113 | 643 | 85 | -15 | 550 | 53 | -- | -- | -- | -- | -- | -- |
| Angelini et al, 2012 – 51 | Myozyme | 0 | 35 | Female | -- | 3 | -- | -- | 28 | -- | 75 | -- | -- | -- | -- | -- | -- | -- |
| Angelini et al, 2012 – 51 | Myozyme | 36 | 35 | Female | -- | 3 | 18 | 156 | 32 | 7 | 32.5 | 89 | -- | -- | -- | -- | -- | -- |
| Angelini et al, 2012 – 52 | Myozyme | 0 | 39 | Female | -- | 34 | -- | -- | 113 | -- | 643 | -- | -- | -- | -- | -- | -- | -- |
| Angelini et al, 2012 – 52 | Myozyme | 36 | 39 | Female | -- | 34 | 61 | 307 | 98 | -11 | 696 | 18 | -- | -- | -- | -- | -- | -- |
| Angelini et al, 2012 – 53 | Myozyme | 0 | 38 | Male | -- | 25 | -- | -- | 18 | -- | 156 | -- | -- | -- | -- | -- | -- | -- |
| Angelini et al, 2012 – 53 | Myozyme | 12 | 38 | Male | -- | 25 | 75 | 368 | 25 | 3 | 245 | 32 | -- | -- | -- | -- | -- | -- |
| Angelini et al, 2012 – 54 | Myozyme | 0 | 54 | Female | -- | 42 | -- | -- | 61 | -- | 307 | -- | -- | -- | -- | -- | -- | -- |
| Angelini et al, 2012 – 55 | Myozyme | 0 | 10 | Male | -- | 3 | -- | -- | 50 | -- | 325 | -- | -- | -- | -- | -- | -- | -- |
| Angelini et al, 2012 – 55 | Myozyme | 36 | 10 | Male | -- | 3 | 88 | 580 | 75 | -3 | 368 | 312 | -- | -- | -- | -- | -- | -- |
| Angelini et al, 2012 – 58 | Myozyme | 0 | 34 | Female | -- | 32 | -- | -- | 78 | -- | 400 | -- | -- | -- | -- | -- | -- | -- |
| Angelini et al, 2012 – 58 | Myozyme | 36 | 34 | Female | -- | 32 | 71 | 423 | 86 | 11 | 260 | 17 | -- | -- | -- | -- | -- | -- |
| Angelini et al, 2012 – 59 | Myozyme | 0 | 50 | Female | -- | 37 | -- | -- | 88 | -- | 580 | -- | -- | -- | -- | -- | -- | -- |
| Angelini et al, 2012 – 59 | Myozyme | 36 | 50 | Female | -- | 37 | 60 | 230 | 85 | -11 | 892 | 65 | -- | -- | -- | -- | -- | -- |
| Angelini et al, 2012 – 60 | Myozyme | 0 | 57 | Female | -- | 50 | -- | -- | 71 | -- | 423 | -- | -- | -- | -- | -- | -- | -- |
| Angelini et al, 2012 – 60 | Myozyme | 12 | 57 | Female | -- | 50 | 79 | 290 | 82 | -27 | 440 | 15 | -- | -- | -- | -- | -- | -- |
| Angelini et al, 2012 – 61 | Myozyme | 0 | 44 | Female | -- | 27 | -- | -- | 60 | -- | 230 | -- | -- | -- | -- | -- | -- | -- |
| Angelini et al, 2012 – 62 | Myozyme | 0 | 35 | Female | -- | 33 | -- | -- | 49 | -- | 295 | -- | -- | -- | -- | -- | -- | -- |
| Angelini et al, 2012 – 62 | Myozyme | 36 | 35 | Female | -- | 33 | 79 | 330 | 79 | 18 | 290 | 60 | -- | -- | -- | -- | -- | -- |
| Angelini et al, 2012 – 64 | Myozyme | 0 | 55 | Female | -- | 55 | -- | -- | 52 | -- | 305 | -- | -- | -- | -- | -- | -- | -- |
| Angelini et al, 2012 – 64 | Myozyme | 24 | 55 | Female | -- | 55 | 69 | 385 | 99 | 15 | 432 | 105 | -- | -- | -- | -- | -- | -- |
| Angelini et al, 2012 – 66 | Myozyme | 0 | 52 | Female | -- | 42 | -- | -- | 79 | -- | 330 | -- | -- | -- | -- | -- | -- | -- |
| Angelini et al, 2012 – 66 | Myozyme | 36 | 52 | Female | -- | 42 | 66 | 153 | 97 | 5 | 390 | 227 | -- | -- | -- | -- | -- | -- |
| Angelini et al, 2012 – 67 | Myozyme | 0 | 60 | Female | -- | 25 | -- | -- | 69 | -- | 385 | -- | -- | -- | -- | -- | -- | -- |
| Angelini et al, 2012 – 68 | Myozyme | 0 | 47 | Male | -- | 43 | -- | -- | 84 | -- | 490 | -- | -- | -- | -- | -- | -- | -- |
| Angelini et al, 2012 – 68 | Myozyme | 36 | 47 | Male | -- | 43 | 65 | 459 | 66 | -3 | 153 | 11 | -- | -- | -- | -- | -- | -- |
| Angelini et al, 2012 – 70 | Myozyme | 0 | 55 | Male | -- | 42 | -- | -- | 71 | -- | 380 | -- | -- | -- | -- | -- | -- | -- |
| Angelini et al, 2012 – 70 | Myozyme | 36 | 55 | Male | -- | 42 | 65 | 480 | 49 | -17 | 256 | -78 | -- | -- | -- | -- | -- | -- |
| Angelini et al, 2012 – 71 | Myozyme | 0 | 50 | Male | -- | 22 | -- | -- | 65 | -- | 459 | -- | -- | -- | -- | -- | -- | -- |
| Angelini et al, 2012 – 71 | Myozyme | 36 | 50 | Male | -- | 22 | 87 | 384 | 62 | -8 | 470 | 20 | -- | -- | -- | -- | -- | -- |
| Angelini et al, 2012 – 72 | Myozyme | 0 | 41 | Female | -- | 30 | -- | -- | 65 | -- | 480 | -- | -- | -- | -- | -- | -- | -- |
| Angelini et al, 2012 – 72 | Myozyme | 36 | 41 | Female | -- | 30 | 75 | 372 | 48 | 6 | 402 | 144 | -- | -- | -- | -- | -- | -- |
| Angelini et al, 2012 – 73 | Myozyme | 0 | 44 | Female | -- | 18 | -- | -- | 87 | -- | 384 | -- | -- | -- | -- | -- | -- | -- |
| Angelini et al, 2012 – 8 | Myozyme | 0 | 35 | Female | -- | 10 | -- | -- | 79 | -- | 404 | -- | -- | -- | -- | -- | -- | -- |
| Angelini et al, 2012 – 8 | Myozyme | 36 | 35 | Female | -- | 10 | 82 | 439 | 75 | -3 | 372 | 45 | -- | -- | -- | -- | -- | -- |
| Angelini et al, 2012 – 9 | Myozyme | 0 | 37 | Male | -- | 10 | -- | -- | 81 | -- | 516 | -- | -- | -- | -- | -- | -- | -- |
| Angelini et al, 2012 – 9 | Myozyme | 18 | 37 | Male | -- | 10 | 53 | 370 | 35 | 3 | 390 | 81 | -- | -- | -- | -- | -- | -- |
| Deroma et al, 2014 – 1 | Myozyme | 0 | 15 | Male | -- | -- | -- | -- | 113 | -- | 643 | -- | -- | -- | -- | -- | -- | -- |
| Deroma et al, 2014 – 1 | Myozyme | 54 | 15 | Male | -- | -- | 113 | 643 | 102 | -11 | 713 | 70 | -- | -- | -- | -- | -- | -- |
| Deroma et al, 2014 – 2 | Myozyme | 0 | 12.5 | Male | -- | -- | -- | -- | 27 | -- | 104 | -- | -- | -- | -- | -- | -- | -- |
| Deroma et al, 2014 – 2 | Myozyme | 54 | 12.5 | Male | -- | -- | 27 | 104 | 24 | -3 | 353 | 249 | -- | -- | -- | -- | -- | -- |
| Deroma et al, 2014 – 3 | Myozyme | 0 | 12.3 | Male | -- | -- | -- | -- | 138 | -- | 617 | -- | -- | -- | -- | -- | -- | -- |
| Deroma et al, 2014 – 3 | Myozyme | 54 | 12.3 | Male | -- | -- | 138 | 617 | 119 | -19 | 821 | 204 | -- | -- | -- | -- | -- | -- |
| Deroma et al, 2014 – 5 | Myozyme | 0 | 10.3 | Male | -- | -- | -- | -- | 88 | -- | 580 | -- | -- | -- | -- | -- | -- | -- |
| Deroma et al, 2014 – 5 | Myozyme | 54 | 10.3 | Male | -- | -- | 88 | 580 | 87 | -1 | 830 | 250 | -- | -- | -- | -- | -- | -- |
| Deroma et al, 2014 – 6 | Myozyme | 0 | 11.5 | Male | -- | -- | -- | -- | 83 | -- | 690 | -- | -- | -- | -- | -- | -- | -- |
| Deroma et al, 2014 – 6 | Myozyme | 48 | 11.5 | Male | -- | -- | 83 | 690 | 91 | 8 | 782 | 92 | -- | -- | -- | -- | -- | -- |
| Deroma et al, 2014 – 7 | Myozyme | 0 | 9.58 | Female | -- | -- | -- | -- | 75 | -- | 636 | -- | -- | -- | -- | -- | -- | -- |
| Deroma et al, 2014 – 7 | Myozyme | 54 | 9.58 | Female | -- | -- | 75 | 636 | 91 | 16 | 641 | 5 | -- | -- | -- | -- | -- | -- |
| Deroma et al, 2014 – 8 | Myozyme | 0 | 7.16 | Female | -- | -- | -- | -- | 93 | -- | 572 | -- | -- | -- | -- | -- | -- | -- |
| Deroma et al, 2014 – 8 | Myozyme | 54 | 7.16 | Female | -- | -- | 93 | 572 | 111 | 18 | 600 | 28 | -- | -- | -- | -- | -- | -- |
| Hundsberger et al, 2014 – 1 | Myozyme | 54 | -- | Female | -- | 67 | -- | -- | 62.1 | -- | 415 | -- | -- | -- | -- | -- | -- | -- |
| Hundsberger et al, 2014 – 1 | Myozyme | 54 | -- | Female | -- | 67 | -- | -- | 78 | -- | 311 | -- | -- | -- | -- | -- | -- | -- |
| Hundsberger et al, 2014 – 1 | Myozyme | 6 | -- | Female | -- | 67 | -- | -- | 55 | -- | 311 | -- | -- | -- | -- | -- | -- | -- |
| Hundsberger et al, 2014 – 2 | Myozyme | 0 | -- | Female | -- | 38 | -- | -- | 87 | -- | 506 | -- | -- | -- | -- | -- | -- | -- |
| Hundsberger et al, 2014 – 2 | Myozyme | 24 | -- | Female | -- | 38 | 87 | 506 | 103 | 16 | 584 | 78 | -- | -- | -- | -- | -- | -- |
| Hundsberger et al, 2014 – 2 | Myozyme | 36 | -- | Female | -- | 38 | 87 | 506 | 87.1 | 9.99 | 506 | 0 | -- | -- | -- | -- | -- | -- |
| Hundsberger et al, 2014 – 2 | Myozyme | 48 | -- | Female | -- | 38 | 87 | 506 | 90 | 3 | 569 | 63 | -- | -- | -- | -- | -- | -- |
| Hundsberger et al, 2014 – 3 | Myozyme | 0 | -- | Female | -- | 32 | -- | -- | 97 | -- | 554 | -- | -- | -- | -- | -- | -- | -- |
| Hundsberger et al, 2014 – 3 | Myozyme | 18 | -- | Female | -- | 32 | 97 | 554 | 84.7 | -12 | 571 | 17 | -- | -- | -- | -- | -- | -- |
| Hundsberger et al, 2014 – 3 | Myozyme | 24 | -- | Female | -- | 32 | 97 | 554 | 88.8 | -8.2 | 569 | 15 | -- | -- | -- | -- | -- | -- |
| Hundsberger et al, 2014 – 5 | Myozyme | 24 | -- | Male | -- | 45 | -- | -- | 87.1 | -- | 556 | -- | -- | -- | -- | -- | -- | -- |
| Hundsberger et al, 2014 – 5 | Myozyme | 36 | -- | Male | -- | 45 | -- | -- | 68 | -- | 495 | -- | -- | -- | -- | -- | -- | -- |
| Hundsberger et al, 2014 – 5 | Myozyme | 48 | -- | Male | -- | 45 | -- | -- | 75.1 | -- | 530 | -- | -- | -- | -- | -- | -- | -- |
| Hundsberger et al, 2014 – 6 | Myozyme | 18 | -- | Male | -- | 46 | -- | -- | 91.1 | -- | 540 | -- | -- | -- | -- | -- | -- | -- |
| Hundsberger et al, 2014 – 6 | Myozyme | 24 | -- | Male | -- | 46 | -- | -- | 81.2 | -- | 454 | -- | -- | -- | -- | -- | -- | -- |
| Hundsberger et al, 2014 – 6 | Myozyme | 36 | -- | Male | -- | 46 | -- | -- | 85 | -- | 510 | -- | -- | -- | -- | -- | -- | -- |
| Hundsberger et al, 2014 – 7 | Myozyme | 12 | -- | Female | -- | 38 | -- | -- | 80.1 | -- | 544 | -- | -- | -- | -- | -- | -- | -- |
| Hundsberger et al, 2014 – 7 | Myozyme | 24 | -- | Female | -- | 38 | -- | -- | 60.1 | -- | 504 | -- | -- | -- | -- | -- | -- | -- |
| Hundsberger et al, 2014 – 7 | Myozyme | 3 | -- | Female | -- | 38 | -- | -- | 69.1 | -- | 506 | -- | -- | -- | -- | -- | -- | -- |
| Montagnese et al, 2015 -- 1 | Myozyme | 0 | 66 | -- | -- | 60 | -- | -- | 48 | -- | 283 | -- | -- | -- | -- | -- | -- | -- |
| Montagnese et al, 2015 -- 1 | Myozyme | 24 | 66 | -- | -- | 60 | 89 | 40 | 46 | -24 | 322 | -7 | -- | -- | -- | -- | -- | -- |
| Montagnese et al, 2015 -- 11 | Myozyme | 0 | 61 | -- | -- | 42 | -- | -- | 44 | -- | 334 | -- | -- | -- | -- | -- | -- | -- |
| Montagnese et al, 2015 -- 11 | Myozyme | 12 | 61 | -- | -- | 42 | 69 | 180 | 44 | -6 | 334 | 13 | -- | -- | -- | -- | -- | -- |
| Montagnese et al, 2015 -- 12 | Myozyme | 0 | 36 | -- | -- | 34 | -- | -- | 43 | -- | 320 | -- | -- | -- | -- | -- | -- | -- |
| Montagnese et al, 2015 -- 12 | Myozyme | 12 | 36 | -- | -- | 34 | 82 | 445 | 40 | 4 | 319 | 17 | -- | -- | -- | -- | -- | -- |
| Montagnese et al, 2015 -- 13 | Myozyme | 0 | 42 | -- | -- | 34 | -- | -- | 41 | -- | 260 | -- | -- | -- | -- | -- | -- | -- |
| Montagnese et al, 2015 -- 13 | Myozyme | 48 | 42 | -- | -- | 34 | 70 | 310 | 40 | -13 | 271 | -3 | -- | -- | -- | -- | -- | -- |
| Montagnese et al, 2015 -- 15 | Myozyme | 0 | 65 | -- | -- | 56 | -- | -- | 38 | -- | 260 | -- | -- | -- | -- | -- | -- | -- |
| Montagnese et al, 2015 -- 15 | Myozyme | 36 | 65 | -- | -- | 56 | 86 | 470 | 40 | -5 | 258 | -33 | -- | -- | -- | -- | -- | -- |
| Montagnese et al, 2015 -- 19 | Myozyme | 0 | 52 | -- | -- | 52 | -- | -- | 37 | -- | 260 | -- | -- | -- | -- | -- | -- | -- |
| Montagnese et al, 2015 -- 19 | Myozyme | 36 | 52 | -- | -- | 52 | 60 | 380 | 38 | -4 | 255 | 48 | -- | -- | -- | -- | -- | -- |
| Montagnese et al, 2015 -- 2 | Myozyme | 0 | 52 | -- | -- | 52 | -- | -- | 39 | -- | 260 | -- | -- | -- | -- | -- | -- | -- |
| Montagnese et al, 2015 -- 2 | Myozyme | 36 | 52 | -- | -- | 52 | 43 | 450 | 45 | 1 | 273 | 23 | -- | -- | -- | -- | -- | -- |
| Montagnese et al, 2015 -- 28 | Myozyme | 0 | 46 | -- | -- | 46 | -- | -- | 48 | -- | 282 | -- | -- | -- | -- | -- | -- | -- |
| Montagnese et al, 2015 -- 28 | Myozyme | 18 | 46 | -- | -- | 46 | 64 | 430 | 48 | 8 | 291 | 32 | -- | -- | -- | -- | -- | -- |
| Montagnese et al, 2015 -- 3 | Myozyme | 0 | 40 | -- | -- | 38 | -- | -- | 45 | -- | 303 | -- | -- | -- | -- | -- | -- | -- |
| Montagnese et al, 2015 -- 3 | Myozyme | 36 | 40 | -- | -- | 38 | 82 | 365 | 50 | -4 | 323 | -14 | -- | -- | -- | -- | -- | -- |
| Montagnese et al, 2015 -- 5 | Myozyme | 0 | 46 | -- | -- | 40 | -- | -- | 47 | -- | 333 | -- | -- | -- | -- | -- | -- | -- |
| Montagnese et al, 2015 -- 5 | Myozyme | 12 | 46 | -- | -- | 40 | 86 | 390 | 42 | 5.8 | 347 | 23 | -- | -- | -- | -- | -- | -- |
| Montagnese et al, 2015 -- 5 | Myozyme | 24 | 46 | -- | -- | 40 | 86 | 390 | 43 | 1 | 205 | -24 | -- | -- | -- | -- | -- | -- |
| Montagnese et al, 2015 -- 5 | Myozyme | 36 | 46 | -- | -- | 40 | 86 | 390 | 48 | 6.8 | 265 | -32 | -- | -- | -- | -- | -- | -- |
| Montagnese et al, 2015 -- 5 | Myozyme | 48 | 46 | -- | -- | 40 | 86 | 390 | 41 | -6.4 | 265 | -69 | -- | -- | -- | -- | -- | -- |
| Montagnese et al, 2015 -- 5 | Myozyme | 54 | 46 | -- | -- | 40 | 86 | 390 | 44 | -10 | 270 | -100 | -- | -- | -- | -- | -- | -- |
| Montagnese et al, 2015 -- 5 | Myozyme | 6 | 46 | -- | -- | 40 | 86 | 390 | 48 | 3.1 | 273 | 10 | -- | -- | -- | -- | -- | -- |
| Montagnese et al, 2015 -- 7 | Myozyme | 0 | 59 | -- | -- | 59 | -- | -- | 51 | -- | 269 | -- | -- | -- | -- | -- | -- | -- |
| Montagnese et al, 2015 -- 7 | Myozyme | 36 | 59 | -- | -- | 59 | 81 | 88 | 47 | 17 | 297 | 32 | -- | -- | -- | -- | -- | -- |
| Montagnese et al, 2015 -- 8 | Myozyme | 0 | 72 | -- | -- | 64 | -- | -- | 70 | -- | 250 | -- | -- | -- | -- | -- | -- | -- |
| Montagnese et al, 2015 -- 8 | Myozyme | 12 | 72 | -- | -- | 64 | 79.7 | 330 | 70 | -14 | 260 | 55 | -- | -- | -- | -- | -- | -- |
| Montagnese et al, 2015 -- 8 | Myozyme | 24 | 72 | -- | -- | 64 | 79.7 | 330 | 66 | -21 | 268 | 112 | -- | -- | -- | -- | -- | -- |
| Montagnese et al, 2015 -- 8 | Myozyme | 36 | 72 | -- | -- | 64 | 79.7 | 330 | 65 | -23 | 267 | 72 | -- | -- | -- | -- | -- | -- |
| Montagnese et al, 2015 -- 8 | Myozyme | 48 | 72 | -- | -- | 64 | 79.7 | 330 | 69 | -14 | 257 | 3 | -- | -- | -- | -- | -- | -- |
| Montagnese et al, 2015 -- 8 | Myozyme | 54 | 72 | -- | -- | 64 | 79.7 | 330 | 65 | -11 | 257 | 18 | -- | -- | -- | -- | -- | -- |
| Montagnese et al, 2015 -- 8 | Myozyme | 6 | 72 | -- | -- | 64 | 79.7 | 330 | 63 | -5 | 253 | 18 | -- | -- | -- | -- | -- | -- |
| Patel et al, 2012 -- 2 | Myozyme | 0 | 56 | Female | -- | 49 | -- | -- | 48 | -- | 80 | -- | 44.1 | -- | -- | -- | -- | -- |
| Patel et al, 2012 -- 2 | Myozyme | 12 | 56 | Female | -- | 49 | 103 | 300 | 58 | 5 | 120 | 15 | 48.1 | 4 | -- | -- | -- | -- |
| Patel et al, 2012 -- 2 | Myozyme | 24 | 56 | Female | -- | 49 | 103 | 300 | 52 | -5.6 | 127 | 30 | 46.2 | 2.1 | -- | -- | -- | -- |
| Patel et al, 2012 -- 2 | Myozyme | 6 | 56 | Female | -- | 49 | 103 | 300 | 55 | 5 | 131 | 0 | -- | -- | -- | -- | -- | -- |
| Patel et al, 2012 -- 3 | Myozyme | 0 | 57 | Female | -- | 41 | -- | -- | 60 | -- | 133 | -- | 32.1 | -- | -- | -- | -- | -- |
| Patel et al, 2012 -- 3 | Myozyme | 3 | 57 | Female | -- | 41 | 72 | 191 | 59 | -1 | 160 | -11 | 32.1 | 0 | -- | -- | -- | -- |
| Patel et al, 2012 -- 3 | Myozyme | 9 | 57 | Female | -- | 41 | 72 | 191 | 47 | 1 | 360 | -11 | 47 | 14.9 | -- | -- | -- | -- |
| Van Capelle et al, 2008 -- 1 | Myozyme | 0 | 5.9 | -- | -- | 3.5 | -- | -- | 56 | -- | 367 | -- | -- | -- | -- | -- | -- | -- |
| Van Capelle et al, 2008 -- 1 | Myozyme | 36 | 5.9 | -- | -- | 3.5 | 94 | 340 | 54 | -2 | 366 | 190 | -- | -- | -- | -- | -- | -- |
| Van Capelle et al, 2008 -- 2 | Myozyme | 0 | 12.7 | -- | -- | 11.6 | -- | -- | 50 | -- | 360 | -- | -- | -- | -- | -- | -- | -- |
| Van Capelle et al, 2008 -- 2 | Myozyme | 36 | 12.7 | -- | -- | 11.6 | 71 | 470 | 51 | 2 | 356 | 110 | -- | -- | -- | -- | -- | -- |
| Van Capelle et al, 2008 -- 3 | Myozyme | 0 | 8.9 | -- | -- | 1.1 | -- | -- | 46 | -- | 357 | -- | -- | -- | -- | -- | -- | -- |
| Van Capelle et al, 2008 -- 3 | Myozyme | 36 | 8.9 | -- | -- | 1.1 | 100 | 520 | 45 | 4 | 327 | 70 | -- | -- | -- | -- | -- | -- |
| Van Capelle et al, 2008 -- 4 | Myozyme | 0 | 12.9 | -- | -- | 3 | -- | -- | 66 | -- | 278 | -- | -- | -- | -- | -- | -- | -- |
| Van Capelle et al, 2008 -- 4 | Myozyme | 36 | 12.9 | -- | -- | 3 | 66 | 400 | 61 | 8 | 293 | 170 | -- | -- | -- | -- | -- | -- |
| Van Capelle et al, 2008 -- 5 | Myozyme | 0 | 15.2 | -- | -- | 2 | -- | -- | 61 | -- | 280 | -- | -- | -- | -- | -- | -- | -- |
| Van Capelle et al, 2008 -- 5 | Myozyme | 36 | 15.2 | -- | -- | 2 | 57 | 550 | 63 | 19 | 289 | 100 | -- | -- | -- | -- | -- | -- |
| Vielhaber et al, 2011 -- 1 | Myozyme | 0 | 41 | Male | -- | 30 | -- | -- | 58 | -- | 280 | -- | -- | -- | -- | -- | -- | -- |
| Vielhaber et al, 2011 -- 1 | Myozyme | 12 | 41 | Male | -- | 30 | 81 | 387 | 58 | -4 | 269 | 34 | -- | -- | -- | -- | -- | -- |
| Vielhaber et al, 2011 -- 1 | Myozyme | 18 | 41 | Male | -- | 30 | 81 | 387 | 62 | -2 | 281 | 4 | -- | -- | -- | -- | -- | -- |
| Vielhaber et al, 2011 -- 1 | Myozyme | 24 | 41 | Male | -- | 30 | 81 | 387 | 66 | -1 | 360 | 18 | -- | -- | -- | -- | -- | -- |
| Vielhaber et al, 2011 -- 1 | Myozyme | 6 | 41 | Male | -- | 30 | 81 | 387 | 68 | -6 | 370 | 38 | -- | -- | -- | -- | -- | -- |
| Vielhaber et al, 2011 -- 2 | Myozyme | 0 | 42 | Female | -- | 35 | -- | -- | 66 | -- | 385 | -- | -- | -- | -- | -- | -- | -- |
| Vielhaber et al, 2011 -- 2 | Myozyme | 12 | 42 | Female | -- | 35 | 94 | 300 | 66 | -5 | 380 | 70 | -- | -- | -- | -- | -- | -- |
| Vielhaber et al, 2011 -- 2 | Myozyme | 18 | 42 | Female | -- | 35 | 94 | 300 | 69 | 0 | 385 | 76 | -- | -- | -- | -- | -- | -- |
| Vielhaber et al, 2011 -- 2 | Myozyme | 24 | 42 | Female | -- | 35 | 94 | 300 | 65 | -2 | 360 | 75 | -- | -- | -- | -- | -- | -- |
| Vielhaber et al, 2011 -- 2 | Myozyme | 6 | 42 | Female | -- | 35 | 94 | 300 | 64 | 3 | 364 | 67 | -- | -- | -- | -- | -- | -- |

*FVC: forced vital capacity; 6MWT: six-minute walking test; SF-36 PCS: short-form survey with 36 items – physical component score; MIP: maximal inspiratory pressure; MEP: maximal expiratory pressure.*

*.*

**Study Quality**

In this appendix, we present the study quality assessment that was applied to the current evidence base.  **Table 7** and **Table 8** outline the criteria for the two instruments (Cochrane Risk of Bias Assessment and Downs and Black, respectively) to measure study quality. These are followed by the assessment for each included study. **The LOTS trial was deemed to have low risk of bias for all but two of the following criteria in Table 7.** The exceptions were allocation concealment, which was unclear, and other, which was also unclear. The latter was based on the fact that analyses were conducted by the study sponsors.

T**able 7: The Cochrane Collaboration’s tool for assessing risk of bias (Higgins and Green, 2011)**

| Domain | Support for judgement | Review authors’ judgement |
| --- | --- | --- |
| *Selection bias* |  |  |
| Random sequence generation. | Describe the method used to generate the allocation sequence in sufficient detail to allow an assessment of whether it should produce comparable groups. | Selection bias (biased allocation to interventions) due to inadequate generation of a randomised sequence. |
| Allocation concealment. | Describe the method used to conceal the allocation sequence in sufficient detail to determine whether intervention allocations could have been foreseen in advance of, or during, enrolment. | Selection bias (biased allocation to interventions) due to inadequate concealment of allocations prior to assignment. |
| *Performance bias* |  |  |
| Blinding of participants and personnel  *Assessments should be made for each main outcome (or class of outcomes).* | Describe all measures used, if any, to blind study participants and personnel from knowledge of which intervention a participant received. Provide any information relating to whether the intended blinding was effective. | Performance bias due to knowledge of the allocated interventions by participants and personnel during the study. |
| *Detection bias* |  |  |
| Blinding of outcome assessment  *Assessments should be made for each main outcome (or class of outcomes)*. | Describe all measures used, if any, to blind outcome assessors from knowledge of which intervention a participant received. Provide any information relating to whether the intended blinding was effective. | Detection bias due to knowledge of the allocated interventions by outcome assessors. |
| *Attrition bias* |  |  |
| Incomplete outcome data  *Assessments should be made for each main outcome (or class of outcomes).* | Describe the completeness of outcome data for each main outcome, including attrition and exclusions from the analysis. State whether attrition and exclusions were reported, the numbers in each intervention group (compared with total randomized participants), reasons for attrition/exclusions where reported, and any re-inclusions in analyses performed by the review authors. | Attrition bias due to amount, nature or handling of incomplete outcome data. |
| *Reporting bias* |  |  |
| Selective reporting. | State how the possibility of selective outcome reporting was examined by the review authors, and what was found. | Reporting bias due to selective outcome reporting. |
| *Other bias* |  |  |
| Other sources of bias. | State any important concerns about bias not addressed in the other domains in the tool.  If particular questions/entries were pre-specified in the review’s protocol, responses should be provided for each question/entry. | Bias due to problems not covered elsewhere in the table. |

Table 8: Downs and Black instrument to assess methodological quality of randomized and non-randomized studies (Downs and Black, 1998).

| **Reporting** |
| --- |
| 1. *Is the hypothesis/aim/objective of the study clearly described?* |
| *2. Are the main outcomes to be measured clearly described in the Introduction or Methods section?*  If the main outcomes are first mentioned in the Results section, the question should be answered no. |
| *3. Are the characteristics of the patients included in the study clearly described?*  In cohort studies and trials, inclusion and/or exclusion criteria should be given. In case-control studies, a case-definition and the source for controls should be given. |
| *4. Are the interventions of interest clearly described?*  Treatments and placebo (where relevant) that are to be compared should be clearly described. |
| *5. Are the distributions of principal confounders in each group of subjects to be compared clearly described?*  A list of principal confounders is provided. |
| *6. Are the main findings of the study clearly described?*  Simple outcome data (including denominators and numerators) should be reported for all major findings so that the reader can check the major analyses and conclusions. |
| *7. Does the study provide estimates of the random variability in the data for the main outcomes?*  In non-normally distributed data the inter-quartile range of results should be reported. In normally distributed data the standard error, standard deviation or confidence intervals should be reported. If the distribution of the data is not described, it must be assumed that the estimates used were appropriate and the question should be answered yes. |
| *8. Have all important adverse events that may be a consequence of the intervention been reported?*  This should be answered yes if the study demonstrates that there was a comprehensive attempt to measure adverse events. (A list of possible adverse events is provided). |
| *9. Have the characteristics of patients lost to follow-up been described?*  This should be answered yes where there were no losses to follow-up or where losses to follow-up were so small that findings would be unaffected by their inclusion. This should be answered no where a study does not report the number of patients lost to follow-up. |
| *10. Have actual probability values been reported (e.g. 0.035 rather than <0.05) for the main outcomes except where the probability value is less than 0.001?* |
| **External validity**  All the following criteria attempt to address the representativeness of the findings of the study and whether they may be generalised to the population from which the study subjects were derived. |
| *11. Were the subjects asked to participate in the study representative of the entire population from which they were recruited?*  The study must identify the source population for patients and describe how the patients were selected. Patients would be representative if they comprised the entire source population, an unselected sample of consecutive patients, or a random sample. Random sampling is only feasible where a list of all members of the relevant population exists. Where a study does not report the proportion of the source population from which the patients are derived, the question should be answered as unable to determine. |
| *12. Were those subjects who were prepared to participate representative of the entire population from which they were recruited?*  The proportion of those asked who agreed should be stated. Validation that the sample was representative would include demonstrating that the distribution of the main confounding factors was the same in the study sample and the source population. |
| *13. Were the staff, places, and facilities where the patients were treated, representative of the treatment the majority of patients receive?*  For the question to be answered yes the study should demonstrate that the intervention was representative of that in use in the source population. The question should be answered no if, for example, the intervention was undertaken in a specialist center unrepresentative of the hospitals most of the source population would attend. |
| **Internal validity – bias** |
| *14. Was an attempt made to blind study subjects to the intervention they have received?*  For studies where the patients would have no way of knowing which intervention they received, this should be answered yes. |
| *15. Was an attempt made to blind those measuring the main outcomes of the intervention?* |
| *16. If any of the results of the study were based on “data dredging”, was this made clear?*  Any analyses that had not been planned at the outset of the study should be clearly indicated. If no retrospective unplanned subgroup analyses were reported, then answer yes. |
| *17. In trials and cohort studies, do the analyses adjust for different lengths of follow-up of patients, or in case-control studies, is the time period between the intervention and outcome the same for cases and controls?*  Where follow-up was the same for all study patients the answer should yes. If different lengths of follow-up were adjusted for by, for example, survival analysis the answer should be yes. Studies where differences in follow-up are ignored should be answered no. |
| *18. Were the statistical tests used to assess the main outcomes appropriate?*  The statistical techniques used must be appropriate to the data. For example nonparametric methods should be used for small sample sizes. Where little statistical analysis has been undertaken but where there is no evidence of bias, the question should be answered yes. If the distribution of the data (normal or not) is not described it must be assumed that the estimates used were appropriate and the question should be answered yes. |
| *19. Was compliance with the intervention/s reliable?*  Where there was non-compliance with the allocated treatment or where there was contamination of one group, the question should be answered no. For studies where the effect of any misclassification was likely to bias any association to the null, the question should be answered yes. |
| *20. Were the main outcome measures used accurate (valid and reliable)?*  For studies where the outcome measures are clearly described, the question should be answered yes. For studies which refer to other work or that demonstrates the outcome measures are accurate, the question should be answered as yes. |
| **Internal validity - confounding** |
| *21. Were the patients in different intervention groups (trials and cohort studies) or were the cases and controls (case-control studies) recruited from the same population?*  For example, patients for all comparison groups should be selected from the same hospital. The question should be answered unable to determine for cohort and case-control studies where there is no information concerning the source of patients included in the study. |
| *22. Were study subjects in different intervention groups (trials and cohort studies) or were the cases and controls (case-control studies) recruited over the same period of time?*  For a study which does not specify the time period over which patients were recruited, the question should be answered as unable to determine. |
| *23. Were study subjects randomized to intervention groups?*  Studies which state that subjects were randomized should be answered yes except where method of randomization would not ensure random allocation. For example alternate allocation would score no because it is predictable. |
| *24. Was the randomized intervention assignment concealed from both patients and health care staff until recruitment was complete and irrevocable?*  All non-randomized studies should be answered no. If assignment was concealed from patients but not from staff, it should be answered no. |
| *25. Was there adequate adjustment for confounding in the analyses from which the main findings were drawn?*  This question should be answered no for trials if: the main conclusions of the study were based on analyses of treatment rather than intention to treat; the distribution of known confounders in the different treatment groups was not described; or the distribution of known confounders differed between the treatment groups but was not taken into account in the analyses. In non-randomized studies if the effect of the main confounders was not investigated or confounding was demonstrated but no adjustment was made in the final analyses the question should be answered as no. |
| *26. Were losses of patients to follow-up taken into account?*  If the numbers of patients lost to follow-up are not reported, the question should be answered as unable to determine. If the proportion lost to follow-up was too small to affect the main findings, the question should be answered yes. |

Table 9: Reporting bias and external validity components of the Downs and Black study quality assessment for non-RCT studies reporting IPD

| **Primary Author (Year)** | **Q1** | **Q2** | **Q3** | **Q4** | **Q5** | **Q6** | **Q7** | **Q8** | **Q9** | **Q10** | **Q11** | **Q12** | **Q13** |
| --- | --- | --- | --- | --- | --- | --- | --- | --- | --- | --- | --- | --- | --- |
| Adreassen et al, 2014^43^ | Yes | Yes | Yes | Yes | No | Yes | No | No | UTD | No | Yes | UTD | Yes |
| Angelini et al, 2009^26^ | Yes | Yes | Yes | Yes | No | Yes | Yes | Yes | UTD | Yes | UTD | UTD | Yes |
| Angelini et al, 2012^25^ | Yes | Yes | Yes | Yes | No | Yes | No | Yes | UTD | Yes | Yes | UTD | Yes |
| Deroma et al 2014^15^ | Yes | Yes | Yes | Yes | No | Yes | Yes | No | UTD | UTD | No | UTD | Yes |
| Gaeta et al 2013^16^ | Yes | Yes | Yes | UTD | No | Yes | Yes | UTD | No | UTD | Yes | UTD | Yes |
| Hundsberger et al 2014^17^ | Yes | Yes | No | Yes | Yes | Yes | UTD | No | UTD | UTD | UTD | UTD | Yes |
| Merk et al, 2009^27^ | Yes | Yes | Yes | Yes | No | Yes | No | No | UTD | Yes | Yes | UTD | Yes |
| Montagnese et al 2015^18^ | Yes | Yes | Yes | Yes | No | Yes | Yes | Yes | UTD | Yes | No | UTD | Yes |
| Orlikowski et al, 2011^153^ | Yes | Yes | Yes | Yes | No | Yes | No | Yes | UTD | Yes | Yes | UTD | Yes |
| Papadimas et al, 2011^29^ | Yes | Yes | Yes | Yes | No | Yes | No | No | UTD | Yes | UTD | UTD | Yes |
| Patel et al, 2012^23^ | Yes | Yes | Yes | Yes | No | Yes | No | No | UTD | Yes | Yes | UTD | Yes |
| Van Capelle et al, 2010^45^ | Yes | Yes | Yes | Yes | No | Yes | No | No | UTD | Yes | Yes | UTD | Yes |
| Van Capelle et al, 2010b^46^ | Yes | Yes | Yes | Yes | No | Yes | No | No | UTD | Yes | Yes | UTD | Yes |
| Van Der Beek et al, 2009^47^ | Yes | Yes | Yes | Yes | No | Yes | Yes | Yes | UTD | No | Yes | UTD | Yes |
| Vielhaber et al 2011^20^ | Yes | Yes | Yes | Yes | No | Yes | No | No | UTD | Yes | Yes | UTD | Yes |

*Please refer to Table 8 for the specific questions each column refers to.*

Table 10: Confounding bias and internal validity components of the Downs and Black study quality assessment for non-RCT studies reporting IPD

| **Primary Author (Year)** | **Q14** | **Q15** | **Q16** | **Q17** | **Q18** | **Q19** | **Q20** | **Q21** | **Q22** | **Q23** | **Q24** | **Q25** | **Q26** |
| --- | --- | --- | --- | --- | --- | --- | --- | --- | --- | --- | --- | --- | --- |
| Adreassen et al, 2014^43^ | No | No | UTD | Yes | Yes | Yes | Yes | UTD | No | No | UTD | Yes | NA |
| Angelini et al, 2009^26^ | No | No | UTD | No | Yes | Yes | Yes | Yes | No | No | UTD | Yes | NA |
| Angelini et al, 2012^25^ | No | No | UTD | No | Yes | Yes | Yes | Yes | No | No | UTD | Yes | NA |
| Deroma et al 2014^15^ | No | No | No | No | Yes | Yes | Yes | Yes | No | No | UTD | No | UTD |
| Gaeta et al 2013^16^ | UTD | No | No | No | UTD | Yes | UTD | Yes | No | UTD | UTD | No | UTD |
| Hundsberger et al 2014^17^ | No | No | No | No | Yes | Yes | Yes | Yes | No | No | UTD | Yes | NA |
| Merk et al, 2009^27^ | No | No | UTD | No | Yes | Yes | Yes | Yes | No | No | UTD | Yes | NA |
| Montagnese et al 2015^18^ | No | No | No | No | Yes | Yes | Yes | Yes | No | No | UTD | UTD | Yes |
| Orlikowski et al, 2011^153^ | No | No | UTD | No | Yes | Yes | Yes | UTD | No | No | UTD | Yes | NA |
| Papadimas et al, 2011^29^ | No | No | UTD | No | Yes | Yes | Yes | Yes | No | No | UTD | Yes | NA |
| Patel et al, 2012^23^ | No | No | No | No | Yes | Yes | Yes | UTD | No | No | UTD | Yes | NA |
| Van Capelle et al, 2010^45^ | No | No | UTD | No | Yes | Yes | Yes | UTD | No | No | UTD | Yes | NA |
| Van Capelle et al, 2010b^46^ | No | No | UTD | No | Yes | Yes | Yes | UTD | No | No | UTD | Yes | NA |
| Van Der Beek et al, 2009^47^ | No | No | UTD | No | Yes | Yes | Yes | Yes | No | No | UTD | Yes | NA |
| Vielhaber et al 2011^20^ | No | No | UTD | No | Yes | Yes | Yes | Yes | No | No | UTD | Yes | NA |

*Please refer to Table 8 for the specific questions each column refers to.*

Figure 2: Mean change in (A) six-minute walking test and (B) forced vital capacity across published LOPD studies

**(A)**


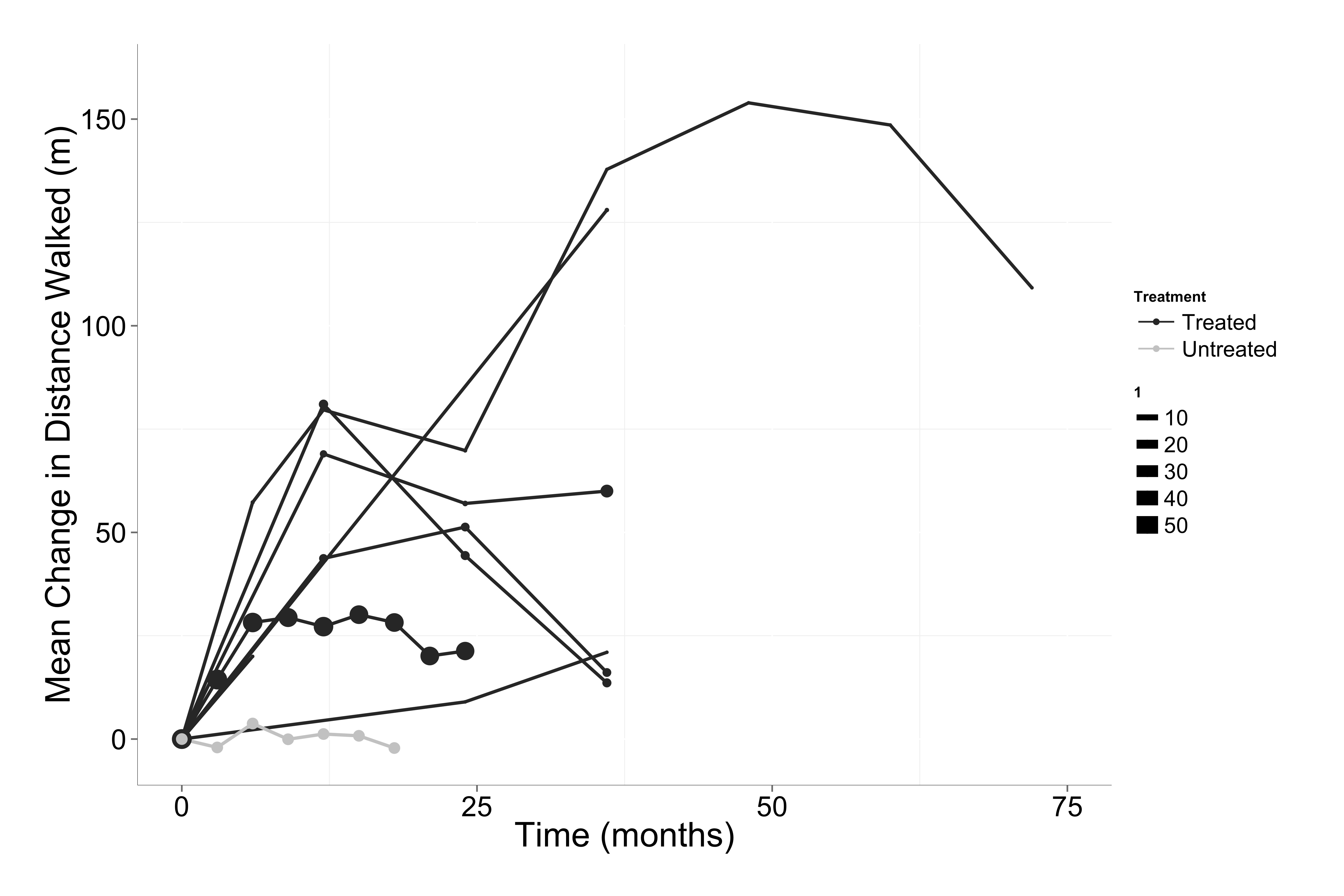


**(B)**


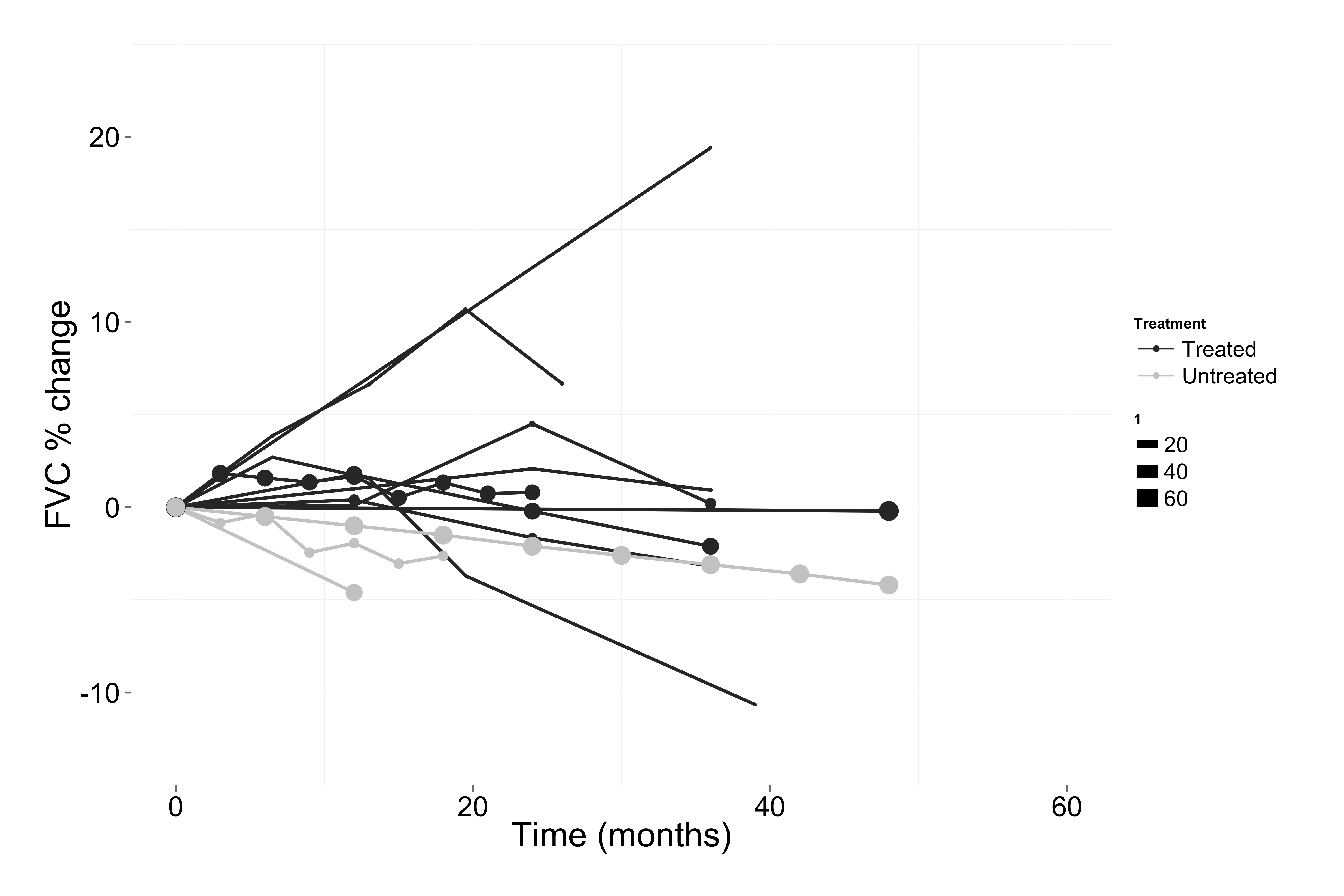


**Figure 3: Association between FVC% and GSCG score**

***Legend:*** *Colors correspond to the studies making up the synthetic cohort. Panel A displays the relationship between the change in GSGC and change in FVC results with a regression line. Panel B displays the relationship between the observed FVC and observed GSGC results with a regression line. Panel C displays the trajectories by individuals across time for change. Panel D displays the trajectories by individuals across time for observed values.*
